# A fish-specific E3 ubiquitin ligase NHRE3 suppresses antiviral immunity by degrading ISG15 via ubiquitination

**DOI:** 10.1371/journal.ppat.1014544

**Published:** 2026-09-29

**Authors:** Juhong Xie, Jiayi Lei, Qinxue Zhu, Bin Gui, Yongming Li, Yaping Wang, Rong Huang

**Affiliations:** 1 Institute of Hydrobiology, Chinese Academy of Sciences, Wuhan, China; 2 College of Advanced Agricultural Sciences, University of Chinese Academy of Sciences, Beijing, China; University of Arkansas for Medical Sciences, UNITED STATES OF AMERICA

## Abstract

Grass carp (*Ctenopharyngodon Idella*) hemorrhagic diseases (GCHD), caused by grass carp reovirus (GCRV), has posed a serious threat to the grass carp aquaculture industry in China. Ubiquitination plays a central role in viral evasion of the host interferon response; therefore, elucidating the mechanisms by which viruses exploit ubiquitination to achieve immune escape is of significant scientific importance and translational value. In a previous study, we identified an E3 ubiquitin ligase–like gene associated with susceptibility to hemorrhagic disease through random mutagenesis in rare minnow (*Gobiocypris rarus*). In this study, domain and amino acid sequence analyses confirmed that this gene encodes a fish-specific E3 ubiquitin ligase, designated NHRE3. *nhre3*^−/−^ rare minnows exhibited significantly enhanced resistance to GCRV. Interferon-stimulated gene 15 (ISG15) was identified as an NHRE3-interacting protein via Immunoprecipitation–Mass Spectrometry (IP–MS). Furthermore, NHRE3 possessed E3 ubiquitin ligase activity, catalyzing K48-linked polyubiquitination of ISG15 and leading to its degradation via the proteasome pathway. Given that the antiviral mechanism of ISG15 in fish remains poorly understood, we further investigated its function and found that ISG15 can interact with IRF3 and promote the expression of innate immune factors downstream of IRF3, thereby exerting its anti-GCRV effect. Taken together, NHRE3 promotes GCRV infection by mediating the ubiquitin–proteasome–dependent degradation of ISG15, may represent a mechanism unique to fish viruses. In mammals, viral immune evasion strategies targeting ISG15 primarily involve the inhibition of ISGylation or induction of deISGylation. The immune evasion mechanism identified in this study, in which a virus promotes degradation of ISG15 via a fish-specific ubiquitin ligase, may represent a more primitive or diverse form of innate immune regulation, revealing the ancient origins of the immunological arms race in vertebrates.

## Author summary

Hemorrhagic disease caused by grass carp reovirus (GCRV) infection is a major problem affecting the grass carp (*Ctenopharyngodon Idella*) aquaculture industry. We previously identified the susceptibility gene nhre3 for GCRV. In this study, we generated homozygous rare minnow lines resistant to GCRV by knocking out this gene and conducted a preliminary assessment of their growth and development. These findings provide valuable references and potential targets for breeding grass carp resistant to hemorrhagic disease. Further investigation revealed that NHRE3 is a fish-specific E3 ubiquitin ligase. In subsequent mechanistic studies, we identified ISG15 as an interacting partner of NHRE3 and demonstrated that NHRE3 degrades ISG15 via the K48-linked ubiquitin-proteasome pathway. This represents a mechanism for downregulating ISG15 distinct from that observed in mammals, thereby contributing to our understanding of the evolution of the vertebrate immune system. The function and mechanism of ISG15 in combating GCRV remain unclear. We found that ISG15 can interact with the host IRF3 and promote the expression of downstream innate immune factors to exert its antiviral effect against GCRV. Consequently, we conclude that the fish-specific E3 ubiquitin ligase NHRE3 suppresses antiviral immunity by ubiquitinating and degrading ISG15.

## Introduction

Innate antiviral immunity constitutes the first line of defense against viral infection in the host. Upon viral infection, nearly all cell types induce the expression of type I interferons (IFN-I) and downstream interferon-stimulated genes (ISGs), thereby mediating the antiviral innate immune response [1]. In fish, owing to the inefficiency of adaptive immune responses, innate immunity can respond to pathogens within an extremely short timeframe [2] and consequently plays a more critical role in the early blockade of pathogen invasion [3]. Grass carp hemorrhagic disease (GCHD), caused by infection with grass carp reovirus (GCRV), is highly detrimental, with a mortality rate exceeding 80% in grass carp (*Ctenopharyngodon idella*), resulting in substantial economic losses to the grass carp aquaculture industry. Therefore, studies on fish immunity will contribute to the advancement of this sector. Given the ecological and economic importance of fish, their susceptibility to infectious diseases, and their utility as models for investigating immune system evolution and conducting biomedical research, fish immunology has become an increasingly important field of study.

Ubiquitination is a post-translational protein modification defined as the covalent conjugation of ubiquitin to lysine residues or the N-terminus of target proteins [4]. By regulating the lifespan and activity of key immune proteins, ubiquitination plays an indispensable role in modulating all stages of the immune response, including its initiation, magnitude, duration, and termination [5,6]. Enzymes involved in ubiquitination include ubiquitin-activating enzymes (E1), ubiquitin-conjugating enzymes (E2), ubiquitin ligases (E3), and deubiquitinating enzymes (DUBs) [7]. Among these, substrate selection by E3 ligases is crucial for the specificity of ubiquitination regulation [8]. Based on their distinct structural domains and functions, E3 ligases are classified into four types: homologous to E6-associated protein carboxyl terminus (HECT), really interesting new gene (RING), U-box, and RING–IBR–RING (RBR) [9,10].

Mammalian viruses can exploit E3 ubiquitin ligases to negatively regulate host innate immune factors, thereby promoting immune evasion and viral infection. For instance, the human papillomavirus E6 protein can hijack the ubiquitination activity of the E6AP ubiquitin ligase, leading to an increased rate of degradation of the tumor suppressor protein p53 [11]. Ankyrin repeat and SOCS box protein 3, acting as an E3 ubiquitin ligase, can mediate the degradation of MAVS upon RNA virus infection, subsequently downregulating downstream antiviral signaling pathways and establishing it as a key negative regulator of antiviral signal activation [12]. Viral infection can promote the polyubiquitination and degradation of nuclear IRF3 by TRIM26, thereby negatively regulating the production of IFN-β [13]. Fish viruses similarly utilize host ubiquitin ligases to degrade innate immune factors. For example, GCRV induces the upregulation of the ubiquitin ligase RNF182, which mediates the ubiquitination of RIG-I in grass carp and rare minnow (*Gobiocypris rarus*), thereby negatively regulating innate immune responses [14]. Japanese sea bass (*Lateolabrax japonicus*) RNF34 promotes the evasion of nervous necrosis virus from antiviral innate immunity by targeting TBK1 and IRF3 for ubiquitination and degradation [15]. The zebrafish (*Danio rerio*) F-box protein Fbxo3 negatively regulates the antiviral response by promoting K27-linked polyubiquitination of the transcription factors IRF3 and IRF7 [16]. In summary, ubiquitination mediated by E3 ubiquitin ligases plays a critical role in viral evasion of host innate immunity.

The IFN response constitutes the first line of defense in innate antiviral immunity. For a virus to successfully infect, replicate, and spread, it must first breach this barrier [17,18]. ISGs, whose expression is induced by IFNs, are key effector molecules of IFN signaling. ISG15, the first ISG gene to be identified, interacts with the ubiquitination system to regulate host immunity [19,20]. ISG15 can exert antiviral effects by modifying viral proteins. For example, ISGylation of the influenza A virus non-structural protein 1 (NS1) impairs the packaging of viral ribonucleoproteins (RNPs), thereby inhibiting viral replication and subsequently enhancing the antiviral activity of IFNs [21]. Furthermore, the replication and dissemination of respiratory syncytial virus [22], Human Papillomavirus type 16 [23], Sindbis virus [24], and Ebola virus [25] are modified by ISG15 conjugation. ISG15 can also exert antiviral effects by modifying host proteins. Both type I IFNs and viral infection induce the covalent modification of IRF3 by ISG15, which promotes IRF3 nuclear translocation, binding to the IFNβ promoter, and downstream IFN signaling [26]. Given the critical antiviral role of ISG15, viruses have evolved multiple strategies to counteract its antiviral effects, including inhibition of ISGylation [27,28] or induction of deISGylation [29,30]. For instance, the PLpro protease of SARS-CoV exhibits deISGylating activity towards ISG15 modifications [31,32]. Similarly, Crimean–Congo hemorrhagic fever orthonairovirus, porcine reproductive and respiratory syndrome virus, and equine arteritis virus encode proteases containing OTU domains with properties similar to those of PLpro [33], which can reduce the levels of ubiquitin and ISG15 conjugates within cells. These findings reveal that viruses have developed diverse immune evasion strategies targeting ISG15, a crucial innate immune effector.

We previously used rare minnow as a model for studying grass carp hemorrhagic disease and found that suppressing the expression of a E3 ubiquitin ligase gene, NHRE3, can significantly inhibit GCRV infection [34]. In this study, we demonstrate that NHRE3 interacts with ISG15 and promotes its degradation via the ubiquitin–proteasome pathway, thereby disrupting the interaction between ISG15 and IRF3 and modulating the expression of innate immune factors downstream of IRF3, ultimately promoting GCRV infection. Notably, NHRE3 is a fish-specific E3 ubiquitin ligase that may facilitate immune evasion unique to fish viruses by negatively regulating the IFN response. As lower vertebrates, fish and their immune systems serve as essential models for investigating the evolution of innate immunity in vertebrates. Collectively, our findings reveal a previously unrecognized link in vertebrate immune system evolution, offering a unique perspective on host–pathogen co-evolution.

## Results

### A E3 ubiquitin ligase gene identified through whole-genome random knockout in rare minnow

The rare minnow is an ideal model for studying GCHD. Previous research involving whole-genome random knockout screening in rare minnows identified 23 genes associated with susceptibility to GCHD. Preliminary in vitro assays revealed that knockdown of the susceptibility gene *evm.TU.LG18.404* significantly inhibited GCRV infection [34]. Homology alignment of the rare minnow *evm.TU.LG18.404* gene against the whole genome of grass carp identified a homologous gene in grass carp that encoded a G2E3-like protein. Phylogenetic tree analysis indicated that NHRE3 proteins in fish are distantly related to those in mammals and share low sequence homology (Fig 1A). Similarity analysis comparing the fish G2E3-like protein with G2E3 protein sequences from other animals (Arthropods, Amphibians, Reptiles, Birds and Mammals) showed that the similarity ranged from 0.75% to 45.76%, indicating substantial divergence (Fig 1B). Subsequent domain comparison analysis revealed that although both fish and other animals possess a HECT domain at the C-terminus, the N-terminus regions differ significantly. In other animals, the N-terminus contains the typical PHD domain characteristic of G2E3 proteins, whereas in fish, the N-terminus features an NHR domain. Notably, the human NEURL family of RING-type ubiquitin ligases also possesses an NHR domain at the N-terminus, but their C-terminus contains a RING domain (Fig 1C). These results indicate that the protein encoded by the grass carp homolog of *evm.TU.LG18.404* does not possess the characteristic features of G2E3 proteins. Instead, it contains a HECT domain, conserved E2 enzyme-binding sites, and E3 ubiquitin ligase catalytic sites (S1 Fig). Therefore, the protein was identified as a fish-specific HECT-type ubiquitin ligase, designated NHRE3.

**Fig 1:**
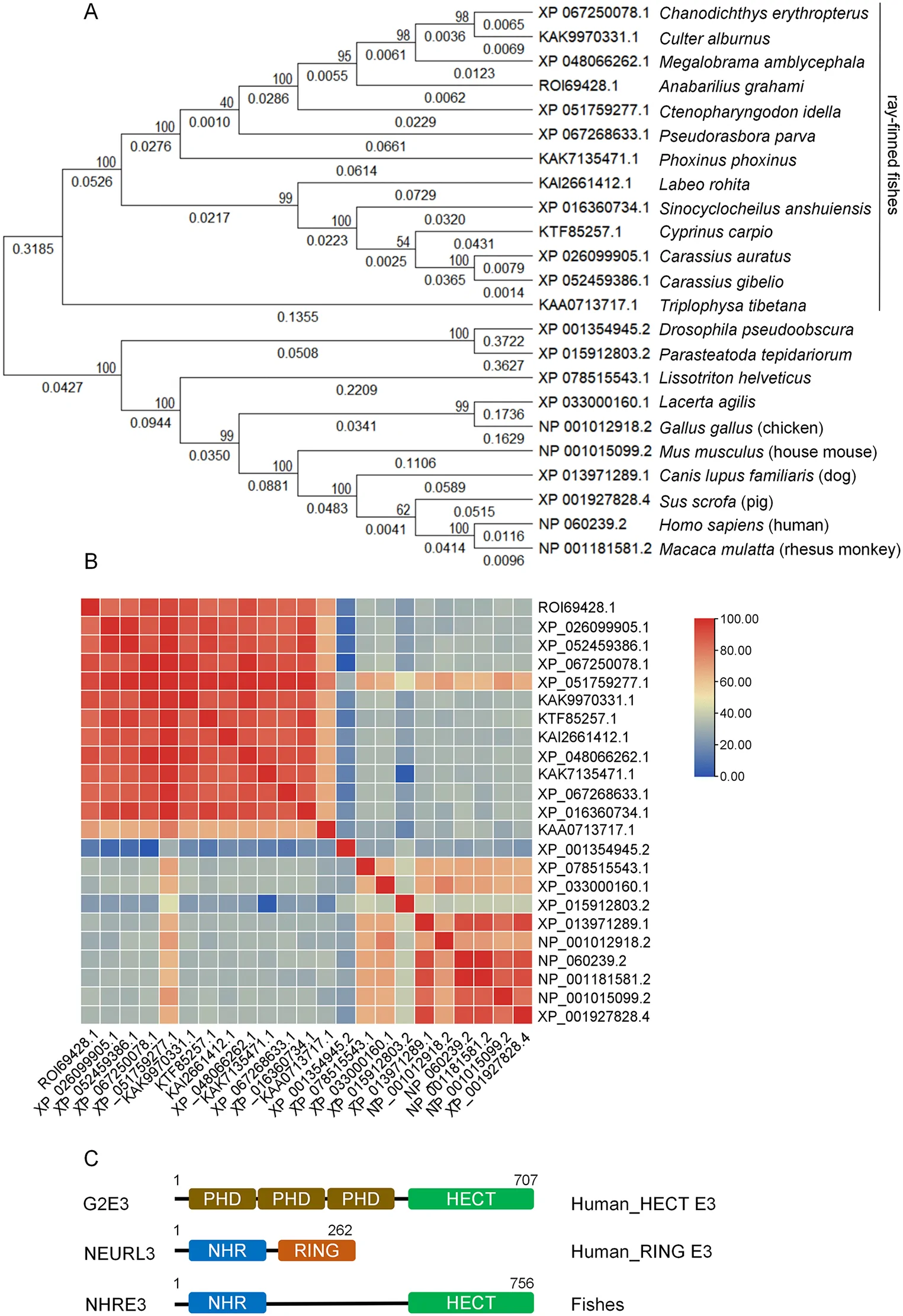
*evm.TU.LG18.404* encodes a fish-specific HECT E3 ubiquitin ligase. (A) Phylogenetic tree of G2E3 protein sequences from fish and other species. The phylogenetic tree was constructed using the neighbor-joining method in MEGA software. The accession numbers for the G2E3 amino acid sequences of each species are as follows: *Chanodichthys erythropterus* XP_067250078.1; *Ctenopharyngodon idella* XP_048066262.1; *Anabarilius grahami* XP_069428.1; *Pseudocoregonus laevis* XP_067250078.1; *A*. *grahami* XP_026099905.1; *Megalobrama amblycephala* XP_048066262.1; *A*. *grahami* ROI69428.1; *Pseudorasbora parva* XP_067268633.1; *Phoxinus phoxinus* KAK7135471.1; *Labeo rohita* KAI2661412.1; *Sinocyclocheilus anshuiensis* XP_016360734.1; *Cyprinus carpio* KTF85257.1; *Carassius auratus* XP_026099905.1; *Carassius gibelio* XP_052459386.1; *Triplophysa tibetana* KAA0713717.1; *Drosophila pseudoobscura* XP_001354945.2; *Parasteatoda tepidariorum* XP_015912803.2; *Lissotriton helveticus* XP_078515543.1; *Lacerta agilis* XP_033000160.1; *Gallus gallus* NP_001012918.2; *Mus musculus* NP_001015099.2; *Canis lupus familiaris* XP_013971289.1; *Sus scrofa* XP_001927828.4; *Homo sapiens* NP_060239.2; *Macaca mulatta* NP_001181581.2. (B) Comparison of G2E3 protein sequence similarity among fish and other species. Sequence similarity is markedly lower in fish than in other species. (C) Comparison of NHRE3, G2E3, and NEULR3 domains. The N-terminus of NHRE3 possesses a unique NHR domain.

### A *nhre3* homozygous mutant of rare minnow exhibits resistance to GCRV

To further ascertain the antiviral function of NHRE3, we generated homozygous NHRE3 knockout mutants in rare minnows. The rare minnow *nhre3* gene comprises 14 exons. We selected a target site within exon 6 and performed targeted *nhre3* gene knockout using CRISPR/Cas9 gene-editing technology. Through Sanger sequencing of the P0, F1, and F2 generations, we screened and identified a mutant line characterized by a 12 bp deletion and a 13 bp insertion compared with that in the wild-type sequence. This line was designated as the *nhre3*^−/−^ strain (Fig 2A). Using qPCR, we further verified the gene knockout effects and found that the expression level of *nhre3* in the *nhre3*^−/−^ strain was significantly reduced compared with that in the wild type (Fig 2B).

**Fig 2:**
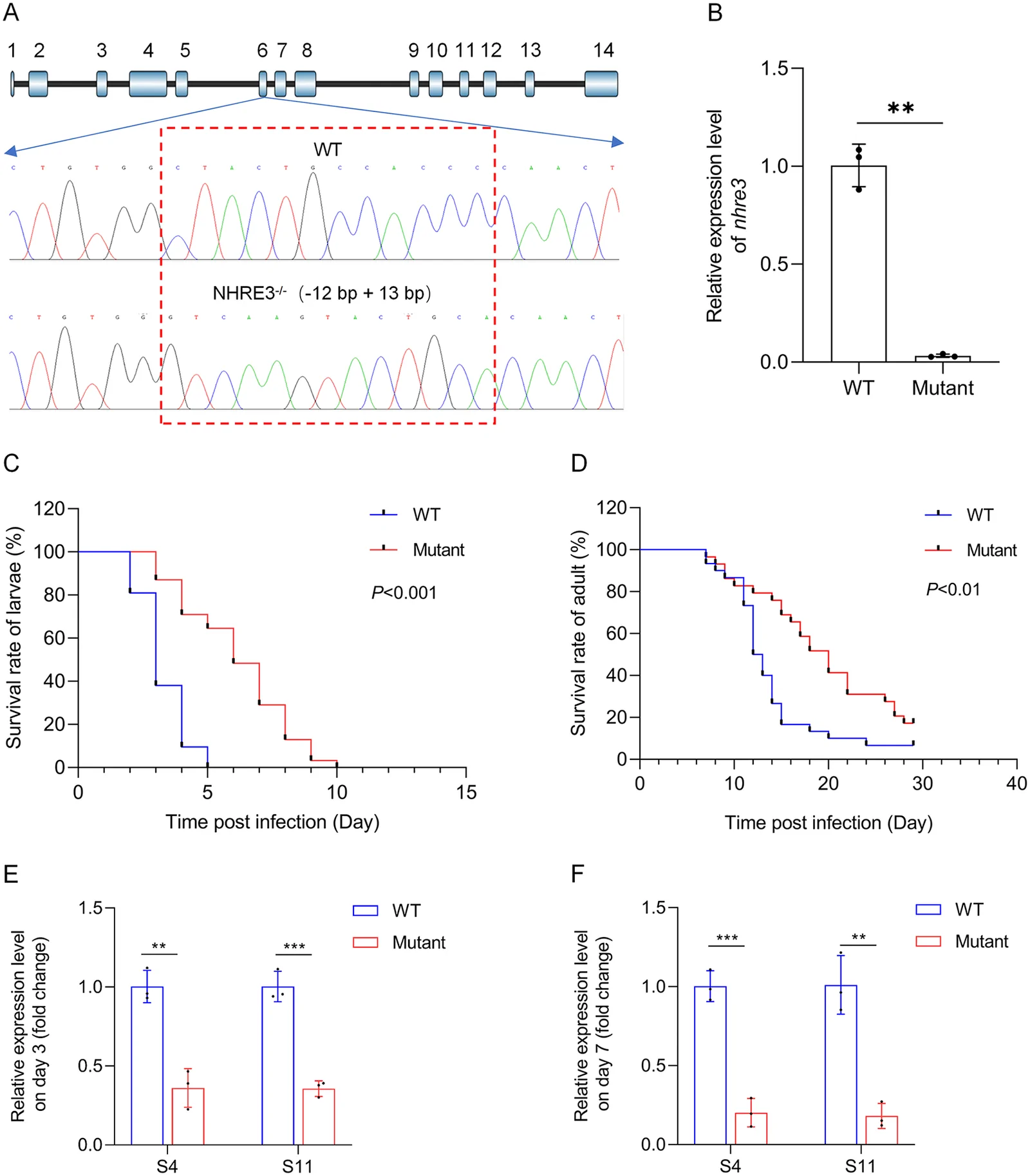
A *nhre3* homozygous mutant of rare minnow exhibits resistance to GCRV. (A) Mutation type in the homozygous *nhre3* knockout mutant of rare minnow. Compared with the wild type, the mutant harbors a 12 bp deletion and a 13 bp insertion within exon 6. (B) Total RNA was extracted from wild-type and mutant larvae, and the expression level of *nhre3* was assessed using qPCR. Expression of *nhre3* in the mutant was significantly reduced compared with that in the wild type. (C) Survival curves of larvae following viral challenge. Mutant larvae exhibited prolonged survival and a reduced mortality rate compared with those in the wild type. (D) Survival curves of adult fish following viral challenge. Mutant adults exhibited prolonged survival and a reduced mortality rate compared with those in the wild type. All experiments were repeated for at least three times with similar results. (E) Expression levels of the GCRV S4 and S11 segments in liver tissue from wild-type and mutant strains on day 4 after infection. (F) Expression levels of the GCRV S4 and S11 segments in liver tissue from wild-type and mutant strains on day 7 after infection.

Subsequently, 20 days post fertilization (dpf) larvae of wild-type (n = 30) and mutant (n = 30) rare minnows were challenged with GCRV using the immersion method, and mortality was recorded at various time points to examine the effects of *nhre3* gene mutations on resistance to GCRV. Wild-type larvae began to die on day 2, with mortality exceeding 50% by day 3, and all larvae had died by day 5. In contrast, mutant larvae began to die on day 3, with mortality exceeding 50% by day 7, and complete mortality was not observed until day 10 (Fig 2C). We further challenged mutant adult fish (n = 29) using the high-salinity immersion method, with wild-type fish (n = 30) serving as controls. The results revealed that wild-type adults began to die on day 7, with mortality exceeding 50% by day 13. Deaths ceased by day 20, with only two surviving fish. In contrast, mutant adults also began to die on day 7, but mortality exceeded 50% by day 18. Deaths ceased by day 28, with five fish ultimately surviving (Fig 2D). In addition, we collected liver tissue from wild-type and mutant rare minnow on days 3 and 7 after viral infection to detect the expression levels of GCRV S4 and S11. The results showed that the viral load in the mutants was significantly lower than that in the wild-type fish (Fig 2E and 2F). Collectively, these results indicate that the loss of *nhre3* enhances resistance to GCRV in rare minnows, identifying *nhre3* as a susceptibility gene for GCRV.

Growth and development rates of fish are important economic indicators that affect aquaculture production. We compared the body morphology of mutant and wild-type larvae and adults, as well as the body length and weight of adult fish, to preliminarily assess the effect of *nhre3* on the growth and development of rare minnows. Comparison of larval and adult body morphology revealed no obvious external differences between mutant and wild-type fish, and development was normal in both sexes. Comparison of body length and weight between mutant and wild-type adult fish showed that the average weight of mutants was slightly higher than that of wild type; however, this difference was not statistically significant (S2 Fig).

### The *nhre3* gene responds to and promotes GCRV infection

The *nhre3* gene is expressed in various tissues of the rare minnow, with expression levels from lowest to highest as follows: kidney, gill, heart, intestine, spleen, liver, skin, brain, and muscle (S3 Fig). To examine the response of *nhre3* to GCRV infection, we collected gill, liver, kidney, and intestinal tissues from rare minnows on days 3 and 7 post-challenge for gene expression analysis. Its expression was significantly upregulated in all examined tissues on day 3 post challenge. On day 7 post-challenge, expression in the gill had returned to pre-challenge levels, whereas it remained upregulated in the other tissues (Fig 3A).

**Fig 3:**
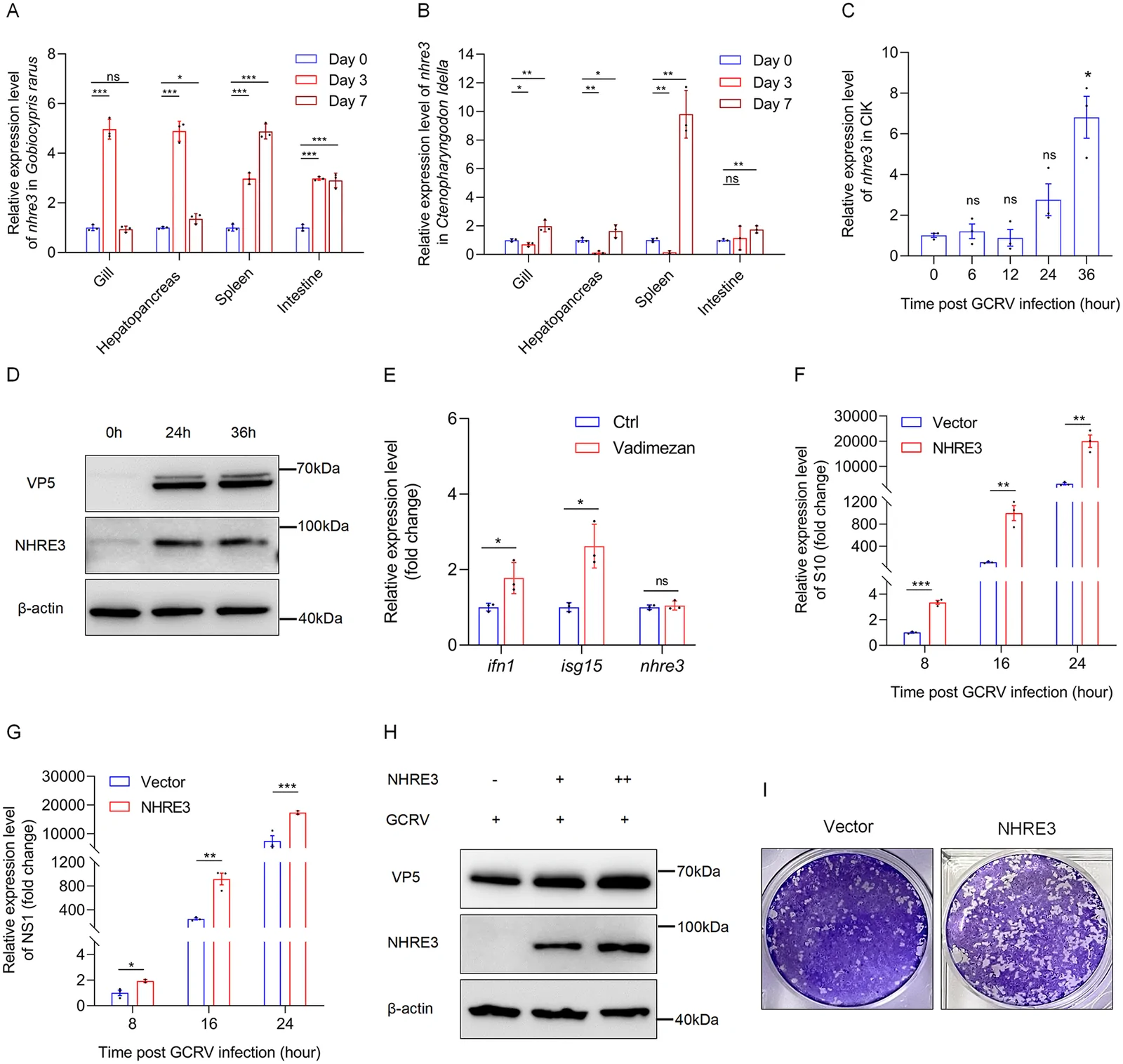
The *nhre3* gene responds to and promotes GCRV infection. (A) qPCR analysis of *nhre3* gene expression in the gill, hepatopancreas, kidney, and intestine of rare minnow on days 0, 3, and 7 post-GCRV infection. (B) qPCR analysis of *nhre3* gene expression in the gill, hepatopancreas, kidney, and intestine of grass carp on days 0, 3, and 7 post-GCRV infection. (C) qPCR analysis of *nhre3* gene expression in CIK cells at 0, 6, 12, 24, and 36 h post-GCRV infection. (D) Western blot analysis of NHRE3 protein and GCRV VP5 protein levels in CIK cells at 0, 6, 12, 24, and 36 h post-GCRV infection. (E) qPCR analysis of *ifn1*, *isg15*, and *nhre3* gene expression levels in CIK cells treated with the activator Vadimezan (100μg/ml) after 24h, DMSO treatment as the control group. (F) CIK cells overexpressing *nhre3* for 24 h were subsequently challenged with GCRV. The expression level of the GCRV S10 segment was measured using qPCR at 8, 16, and 24 h post-challenge. Cells transfected with an empty vector served as the control group. (G) CIK cells overexpressing *nhre3* for 24 h were subsequently challenged with GCRV. The expression level of the GCRV NS1 segment was measured using qPCR at 8, 16, and 24 h post-challenge. Cells transfected with an empty vector served as the control group. (H) CIK cells were transfected with 0, 1, or 2 μg of *nhre3* overexpression plasmid for 24 h and subsequently challenged with GCRV. The expression levels of GCRV VP5 and NHRE3 proteins were measured via western blot at 24 h post-challenge. (I) CIK cells overexpressing *nhre3* for 24 h were subsequently challenged with GCRV. Cytopathic effects (CPE) were assessed using crystal violet staining at 24 h post-challenge. All experiments were repeated for at least three times with similar results.

The rare minnow is a model organism for studying GCRV infection. In aquaculture, GCRV primarily infects grass carp. In grass carp, *nhre3* expression was the highest in the heart and lowest in the brain, with levels lowest to highest as follows: brain, kidney, muscle, intestine, spleen, gill, liver, skin, and heart (S3 Fig). To verify whether the *nhre3* gene in grass carp responds to GCRV infection, we analyzed the changes in nhre3 expression in gill, liver, kidney, and intestinal tissues before and after viral challenge. Its expression was significantly upregulated in all examined tissues on day 7 post-challenge (Fig 3B). Viral challenge experiments in CIK cells demonstrated that *nhre3* expression was significantly upregulated with increasing duration of GCRV infection (Fig 3C). Furthermore, NHRE3 protein levels were also markedly elevated post-challenge (Fig 3D). After treatment with the interferon pathway activator Vadimezan, no significant difference in *nhre3* expression was observed compared to the control group, indicating that *nhre3* is not triggered by the host interferon signaling pathway (Fig 3E). These results indicate that *nhre3* in both rare minnow and grass carp responds to GCRV infection.

Loss of *nhre3* enhances resistance to GCRV in rare minnows. To further investigate the function of *nhre3* in grass carp, we overexpressed grass carp *nhre3* in CIK cells prior to viral challenge and examined the expression of the GCRV S11 and NS1 segment genes. *nhre3* overexpression significantly increased the expression of S10 and NS1 genes over the course of infection (Fig 3F and 3G). Concurrently, GCRV VP5 protein levels were also elevated upon *nhre3* overexpression (Fig 3H). Crystal violet staining demonstrated that *nhre3* overexpression resulted in more extensive cytopathic effects (CPE) following GCRV infection (Fig 3I). Collectively, these results suggest that *nhre3* promotes GCRV infection.

### Grass carp NHRE3 interacts with ISG15

As demonstrated above, *nhre3* is a susceptibility gene for GCRV that responds to and promotes GCRV infection. To investigate the mechanism by which *nhre3* facilitates GCRV infection, we overexpressed *nhre3* in GCO cells, challenged the cells with GCRV as the experimental group, then used IP–MS to screen for proteins interacting with grass carp NHRE3. Cells overexpressing *nhre3* but not infected with GCRV served as the control group, both the experimental group and the control group used the overexpression-empty vector group as a control to screen for candidate interacting proteins. A fold change (FC) > 4 were used as the criteria for screening candidate interacting proteins via IP-MS. A total of 29 candidate interacting proteins were identified in both the control and the experimental groups (Fig 4A and 4B). All proteins identified in the control and experimental groups are listed in S1 Table and S2 Table. A Venn diagram analysis was then performed to compare the proteins identified in both groups. After excluding proteins present in the control group, six candidate interacting proteins associated with GCRV infection were identified in the experimental group (Fig 4C). These six proteins were subsequently annotated as ISG15, NF-κB p65 transcription factor, transcription factor 7, proto-oncogene c-Jun, transcription factor Jun-B, and 40S ribosomal protein LAMR1 (Fig 4D).

**Fig 4:**
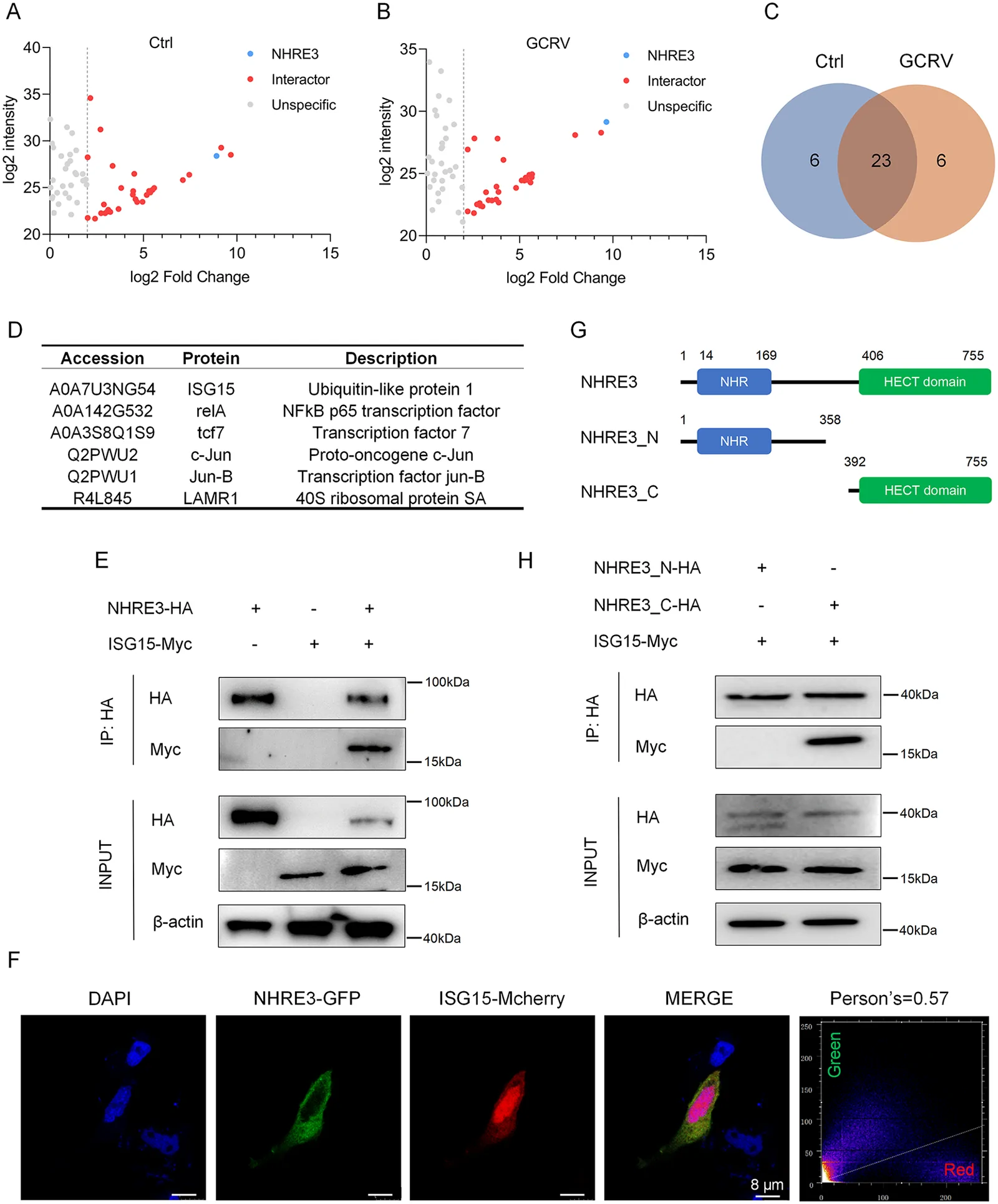
Grass carp NHRE3 interacts with ISG15. (A) Non-specific proteins identified via immunoprecipitation–mass spectrometry (IP–MS) in the control group (NHRE3 overexpression without viral challenge). (B) Candidate interacting proteins identified via IP–MS in the experimental group (NHRE3 overexpression with viral challenge). (C) Venn diagram of proteins identified with IP–MS in the control and experimental groups. After excluding 23 non-specific proteins found in the control group, six candidate interacting proteins were identified in the experimental group. (D) Annotation information for the six candidate interacting proteins. (E) Co-immunoprecipitation (Co-IP) was performed 24 h after co-transfection of pcDNA-NHRE3-HA and pCMV-ISG15-MYC in 293T cells. A distinct band was observed in the IP fraction corresponding to the interaction between NHRE3 and ISG15. (F) Colocalization analysis was performed 24 h after co-transfection of pGFP-NHRE3 and pmCherry-ISG15 in CIK cells. Confocal microscopy revealed cytoplasmic colocalization, which was confirmed by analysis using the ImageJ Colocalization Finder plugin, yielding a Pearson correlation coefficient of 0.57. (G) Domain structure of NHRE3 and construction of truncation mutants. (H) Co-IP was performed 24 h after co-transfection of NHRE3_N and NHRE3_C truncation mutants with pCMV-ISG15-MYC in 293T cells. A distinct band was observed in the IP fraction corresponding to the interaction between the NHRE3_C mutant and ISG15. All experiments were repeated for at least three times with similar results.

ISG15 is an IFN-stimulated factor that plays an important role in innate immunity, and previous studies have demonstrated its antiviral activity [35]. Therefore, we investigated whether NHRE3 interacts with ISG15 using Co-IP and subcellular colocalization analyses. Co-overexpression of NHRE3-HA and ISG15-MYC vectors, followed by IP with HA magnetic beads and detection with an anti-MYC antibody, revealed a distinct band corresponding to ISG15 (Fig 4E). Co-overexpression of NHRE3-GFP and ISG15-mCherry vectors showed that NHRE3 was mainly localized in the cytoplasm, whereas ISG15 was expressed ubiquitously throughout the cell. Colocalization of the two proteins was observed in the cytoplasm (Fig 4F), with a Pearson correlation coefficient of 0.57.

To determine which domain of grass carp NHRE3 interacts with ISG15, we cloned and expressed truncated fragments of NHRE3. The full-length NHRE3 protein consists of 755 amino acids (aa), with the NHR domain located at residues 12–358 and the HECT domain at residues 406–755 (Fig 4G). HA-tagged fusion expression vectors corresponding to each domain were individually co-expressed with the ISG15-MYC vector and subjected to IP using HA magnetic beads. Only the HECT domain was found to interact with ISG15 (Fig 4H). As the HECT domain is a conserved feature of E3 ubiquitin ligases, this finding suggests that NHRE3 may catalyze the ubiquitination of ISG15 by functioning as an E3 ubiquitin ligase.

### Grass carp NHRE3 degrades ISG15 via the K48-linked ubiquitination–proteasome pathway

In CIK cells, a decrease in ISG15 expression is observed after GCRV infection (Fig 5A), suggesting that ISG15 protein levels may undergo degradation induced by GCRV infection. Upon overexpression of NHRE3, the decrease in ISG15 expression was more pronounced than that in the control group (overexpressing the empty vector) (Fig 5B), indicating that the degradation of ISG15 may be mediated by NHRE3. In addition, we measured the expression levels of the *isg15* gene in cells while *nhre3* was overexpressed; the results showed that *nhre3* had no significant effect on ISG15 mRNA expression (Fig 5C), thereby ruling out the possibility that the decrease in ISG15 protein levels was due to transcriptional regulation.

**Fig 5:**
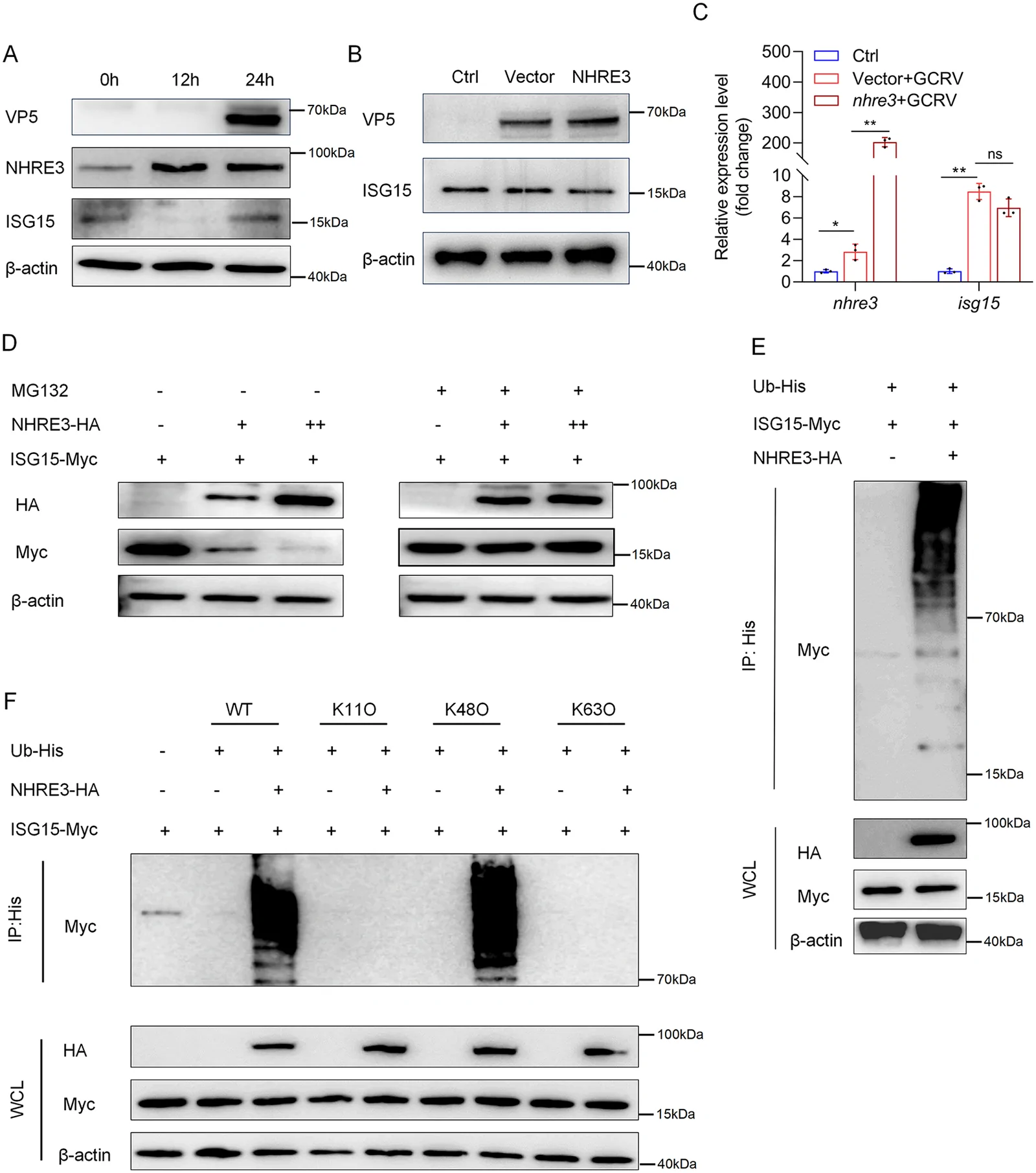
Grass carp NHRE3 degrades ISG15 via the K48-linked ubiquitination–proteasome pathway. (A) Western blot analysis of NHRE3 and ISG15 in CIK cells following GCRV infection, showing ISG degradation. (B) After overexpressing NHRE3, the CIK cells were infected with GCRV, WB was used to detect ISG15 protein expression after 24h, with the uninfected and empty vector overexpression groups serving as controls. (C) After overexpressing *nhre3*, the CIK cells were infected with GCRV, qPCR was used to detect isg15 gene expression after 24h, with the uninfected and empty vector overexpression groups serving as controls. (D) 293T cells were co-transfected with 0, 1, or 2 μg of pcDNA-NHRE3-HA and pCMV-ISG15-MYC. After 18 h, the cells were treated with MG-132 (20 μM) for 6 h, and ISG15 degradation was assessed via western blot. (E) 293T cells were co-transfected with pcDNA-NHRE3-HA, pCMV-ISG15-MYC, and pCMV-Ub-His. ISG15 ubiquitination was assessed using western blot. A control group without pcDNA-NHRE3-HA transfection was included. (F) 293T cells were co-transfected with pcDNA-NHRE3-HA and pCMV-ISG15-MYC in the presence of His-tagged wild-type ubiquitin (WT) or ubiquitin mutants, including K11-only (K11O), K48-only (K48O), and K63-only (K63O). The cells were treated with MG-132 (20 μM) for 6 h, and ISG15 ubiquitination was subsequently analyzed via western blot. A control group without pcDNA-NHRE3-HA transfection was included. All experiments were repeated for at least three times with similar results.

Furthermore, given that the C-terminus of grass carp NHRE3 contains a conserved HECT domain characteristic of E3 ubiquitin ligases, we hypothesized that ISG15 degradation might occur through an NHRE3-mediated ubiquitin–proteasome pathway. To test this, we treated the cells with the proteasome inhibitor MG132. In the control group, ISG15 protein levels decreased in a dose-dependent manner with increasing NHRE3 expression. However, in the experimental group treated with MG132, ISG15 protein levels remained unaffected by NHRE3 expression (Fig 5D). These results indicate that MG132 blocks NHRE3-mediated degradation of ISG15, suggesting that this degradation occurs via the proteasome pathway.

Protein ubiquitination is a critical step in proteasomal degradation. To elucidate the molecular mechanism underlying NHRE3-mediated proteasomal degradation of ISG15, we investigated whether NHRE3 induces the ubiquitination of ISG15 and the type of ubiquitin chain involved. Based on its amino acid structure, we hypothesized that NHRE3 possesses ubiquitin ligase activity. Given that NHRE3 binds to ISG15 and leads to its degradation at the protein level, we further speculated that the effect of NHRE3 on ISG15 may be mediated through ubiquitination. Our ubiquitination assays confirmed this hypothesis, as distinct ubiquitination bands were observed only in the presence of NHRE3 (Fig 5E), indicating that NHRE3 ubiquitinates ISG15. Different types of ubiquitin modifications have distinct biological functions, with K11-, K48-, and K63-linked ubiquitination particularly associated with proteasomal degradation [10]. Using ubiquitin mutants, we found that NHRE3 specifically enhanced K48-linked ubiquitination of ISG15 (Fig 5F). These findings indicate that grass carp NHRE3 catalyzes K48-linked polyubiquitination of ISG15, leading to its subsequent proteasomal degradation.

### Grass carp NHRE3 inhibits ISG15-mediated antiviral function

As NHRE3 degrades ISG15 via the ubiquitin–proteasome pathway and ISG15 is recognized as an antiviral factor, we investigated whether NHRE3 affects ISG15-mediated cellular antiviral responses. In antiviral assays, ISG15 alone inhibited CPE. Conversely, upon co-overexpression of both NHRE3 and ISG15, CPE was significantly increased. (Fig 6A) Consistent with these results, ISG15 alone significantly inhibited the transcription of GCRV gene segments and protein expression of VP5. However, co-overexpression of NHRE3 and ISG15 alleviated the inhibitory effect of ISG15 on GCRV (Fig 6B and 6C). These results indicate that grass carp NHRE3 suppresses the ISG15-mediated antiviral response. Given that the HECT domain of NHRE3 interacts with ISG15, we co-transfected NHRE3 truncation mutants with ISG15, followed by GCRV challenge, and examined the transcription of GCRV gene segments and VP5 protein expression. The NHRE3_C mutant inhibited the antiviral activity of ISG15, whereas the NHRE3_N mutant had no significant effect (Fig 6D and 6E).

**Fig 6:**
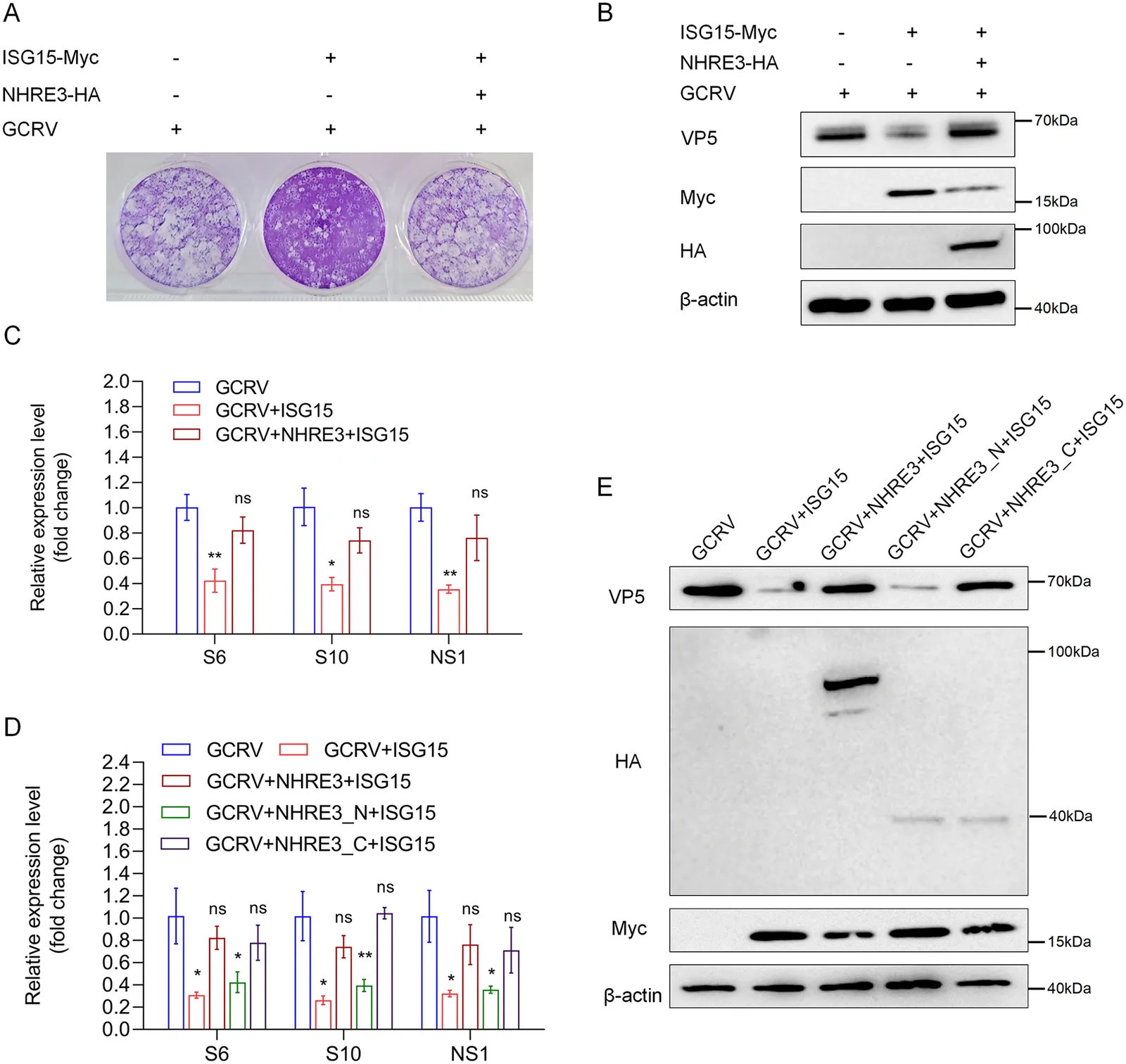
Grass carp NHRE3 inhibits ISG15-mediated antiviral function. (A) CIK cells co-expressing ISG15 and NHRE3 were infected with GCRV. CPE was assessed using crystal violet staining 24 h post-infection. Cells transfected with the empty vector and those transfected with NHRE3 alone served as controls. (B) GCO cells co-expressing ISG15 and NHRE3 were infected with GCRV. The expression level of the GCRV VP5 protein was measured via western blot 24 h post-infection. Cells transfected with the empty vector and those transfected with NHRE3 alone served as controls. (C) GCO cells co-expressing ISG15 and NHRE3 were infected with GCRV. The expression levels of the GCRV S6, S10, and NS1 segments were assessed using qPCR 24 h post-infection. Cells transfected with the empty vector and those transfected with NHRE3 alone served as controls. (D) GCO cells co-expressing ISG15 and different NHRE3 truncation mutants were infected with GCRV. The expression levels of the GCRV S6, S10, and NS1 segments were assessed using qPCR 24 h post-infection. Cells transfected with the empty vector and those transfected with NHRE3 alone served as controls. (E) GCO cells co-expressing ISG15 and different NHRE3 truncation mutants were infected with GCRV. The expression level of the GCRV VP5 protein was measured via western blot 24 h post-infection. Cells transfected with the empty vector and those transfected with NHRE3 alone served as controls. All experiments were repeated for at least three times with similar results.

### ISG15 interacts with IRF3 and promotes the expression of IRF3-regulated downstream immune factors

Mammalian ISG15 exerts antiviral effects by modifying viral proteins. To investigate the anti-GCRV mechanism of grass carp ISG15, we cloned three GCRV gene segments (NS1, NS3, and NS5) and analyzed the interactions between ISG15 and these proteins. ISG15 did not interact with any of these GCRV proteins, suggesting that other viral proteins may be involved (S4 Fig).

Mammalian ISG15 also exerts antiviral effects by modifying the host protein IRF3. Previous studies have shown that grass carp ISG152 can interact with IRF3 [36]. Therefore, we hypothesized that ISG15 may also interact with IRF3. Co-expression of IRF3-HA and ISG15-MYC in cells, followed by Co-IP, revealed an interaction between IRF3 and ISG15. After IP using HA-tag magnetic beads, both the IRF3-HA smear and ISG15-MYC band were detected in the precipitated complex using an anti-HA antibody (Fig 7A). Co-transfection of IRF3-GFP and ISG15-mCherry demonstrated their colocalization in the cytoplasm, with a Pearson correlation coefficient of 0.84 (Fig 7B). IRF3 is a crucial transcription factor in antiviral innate immunity and can directly or indirectly induce the expression of immune factors, such as IFNs, ISGs, and interleukins. We hypothesized that ISG15 might exert its antiviral activity by promoting the expression of immune factors downstream of IRF. Therefore, we overexpressed ISG15 in CIK cells and examined the expression levels of *irf3*, *ifn1*, *ifn3*, *il1β*, *il6*, *il8*, *mx1*, and *mx2* genes. Upon ISG15 overexpression, the expression of *ifn1*, *ifn3*, *il1β*, *il6*, *il8*, and *mx2* was significantly upregulated (Fig 7E–7L), indicating that ISG15 can promote the expression of innate immune factors downstream of IRF3. We simultaneously measured the expression levels of *isg15* and GCRV S6 and confirmed that *isg15* was successfully overexpressed and inhibited GCRV replication (Fig 7C and 7D). Collectively, ISG15 interacts with IRF3 in the cytoplasm and upregulates immune factors downstream of IRF3, thereby contributing to its anti-GCRV effects.

**Fig 7:**
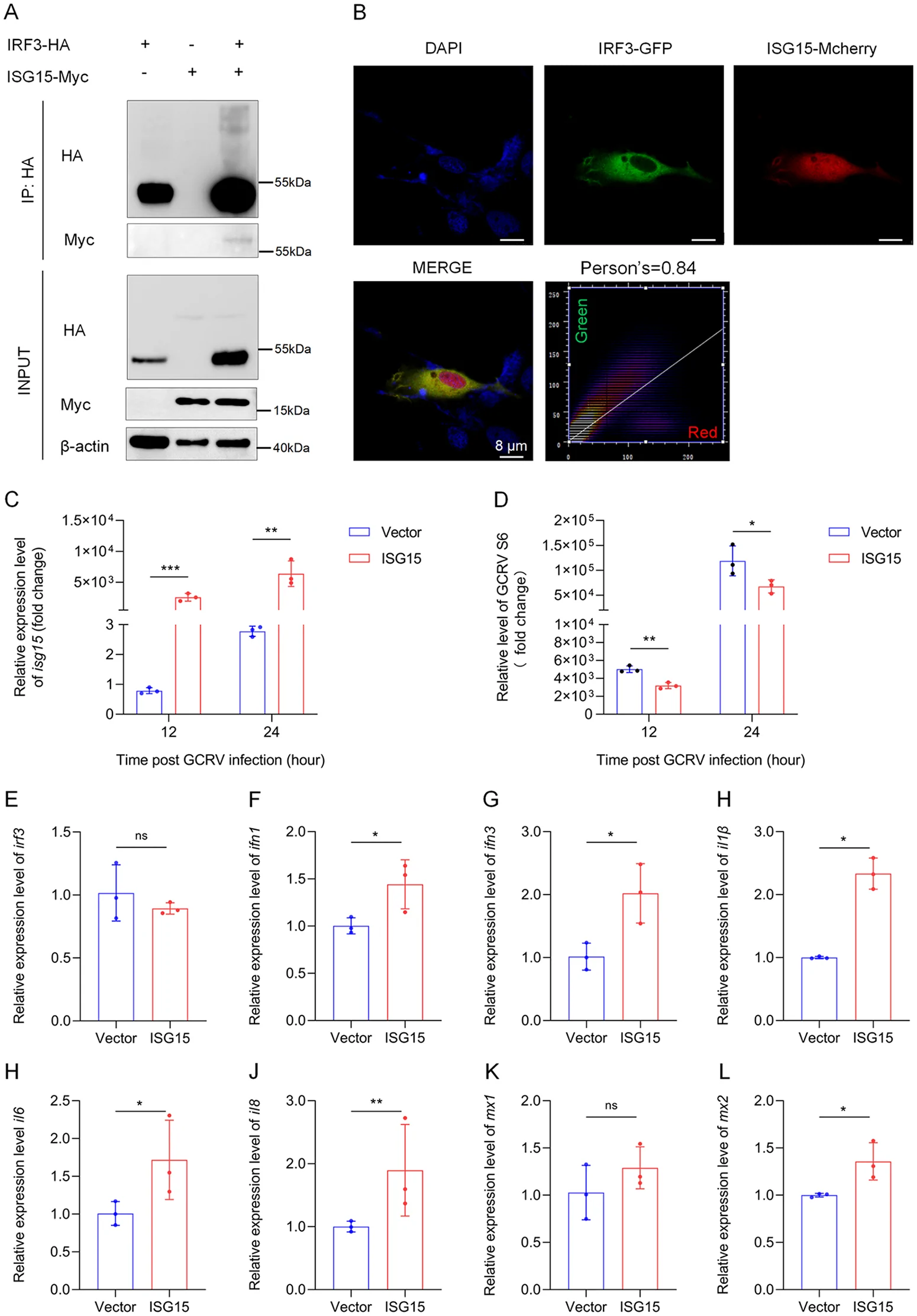
ISG15 interacts with IRF3 and promotes the expression of IRF3-regulated downstream immune factors. (A) Co-expression of ISG15 and IRF3 in GCO cells. Interaction was detected via Co-IP 24 h post-transfection. (B) Co-expression of ISG15-mCherry and IRF3-GFP in GCO cells. Colocalization was observed using a confocal microscope 24 h post-transfection. Analysis using the ImageJ Colocalization Finder plugin confirmed their colocalization, yielding a Pearson correlation coefficient of 0.84. (C) qPCR analysis of *isg15* gene expression levels in CIK cells following ISG15 overexpression. (D) qPCR analysis of GCRV S6 gene expression levels in CIK cells following ISG15 overexpression. (E-L) qPCR analysis of *irf3, ifn1, ifn3, il1β, il6, il8, mx1* and *mx2* gene expression levels in CIK cells following ISG15 overexpression. All experiments were repeated for at least three times with similar results.

## Discussion

In this study, we identified a fish-specific E3 ubiquitin ligase characterized by an N-terminal NHR domain and a C-terminal HECT domain. HECT-type E3 ubiquitin ligases are known to possess a conserved C-terminal HECT domain, whereas their N-terminal domains are complex and diverse [37]. Unlike previously characterized HECT E3 ubiquitin ligases, NHRE3 contains a unique NHR domain at its N-terminus, classifying it as a fish-specific HECT-type E3 ubiquitin ligase. Notably, the NEURL family of RING-type ubiquitin ligases also possesses an N-terminal NHR domain; however, their C-termini contain a RING domain. Studies have shown that NEURL3 deficiency can promote HCV infection [38] and that NEURL3 can act as an E3 ubiquitin ligase for IRF7, positively regulating the antiviral activity of IRF7 [39]. In contrast, this study demonstrates that NHRE3 promotes GCRV infection, suggesting that E3 ubiquitin ligase families in fish and higher mammals follow distinct evolutionary trajectories and may exhibit different functional roles.

*nhre3* is a gene specific to fish, and its expression varies across different tissues in rare minnow and grass carp, with particularly notable differences observed in muscle, brain, and heart tissues. The underlying mechanism may involve mutations in the cis-regulatory elements of gene across different tissues. For example, in mouse and human liver cells, due to mutations in the promoter, when the same transcription factor protein binds to the promoter of a homologous gene, approximately two-thirds of the binding sites do not correspond [40]. This implies that changes in enhancer or promoter sequences can easily lead to significant differences in the expression regulation of the same gene across species.

Previous studies have shown that ISG15, as a ubiquitin-like protein, can conjugate to substrate proteins via ISGylation mediated by E3 ligases such as HERC5, EFP/TRIM25, and ARIH1 [41–45]. In this study, we found that NHRE3 induces ISG15 degradation through the ubiquitin–proteasome pathway rather than catalyzing ISGylation, indicating that NHRE3 does not function as an E3 ligase for ISG15. Viruses can exploit host-encoded E3 ubiquitin ligases to evade innate immunity [46], and fish viruses are also known to utilize host-encoded E3 ubiquitin ligases [14–16]. However, with respect to ubiquitination substrates, mammalian viruses primarily counteract host antiviral responses by antagonizing ISG15 to inhibit ISGylation [27,28] or by inducing deISGylation activity [29,30]. In contrast, this study identified a fish virus that can directly degrade ISG15 by utilizing a host-encoded E3 ubiquitin ligase to evade innate immunity (Fig 8). Because the G2E3 HECT domains of other species show differences in sequence similarity to the NHRE3 HECT domain in fish, and there are currently few reports on the interaction between E3 and ISG15, the interaction between G2E3 and ISG15 in other species requires further research to confirm. In summary, these findings provide new insights into viral strategies targeting ISG15 and may represent a fish virus–specific immune evasion pathway that negatively regulates the IFN response.

**Fig 8:**
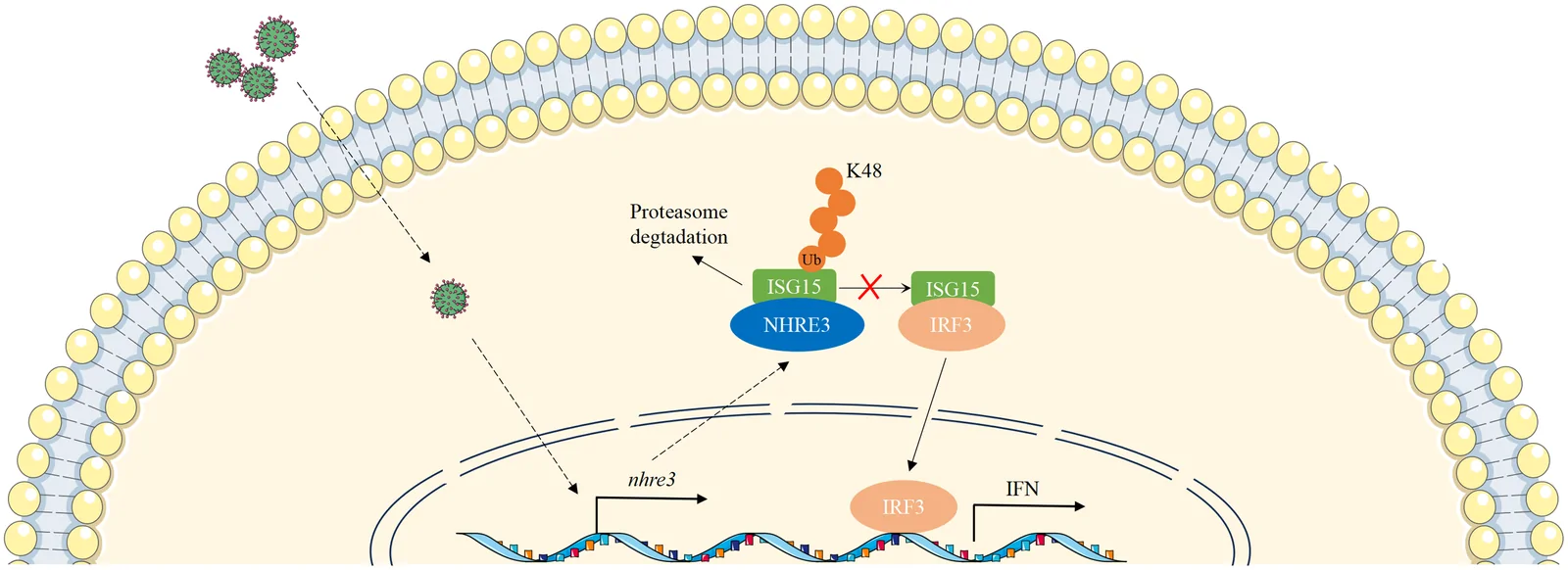
Diagram of the functional mechanism of the GCRV susceptibility gene *nhre3.*

The pathogenicity of various viral infections has long been a focal point of immunological research. ISG15, which has been identified in multiple fish species, is inducible by IFNs [47] and exhibits broad-spectrum antiviral activity. Examples include ISG15 from orange-spotted grouper (*Epinephelus coioides*) against grouper nervous necrosis virus (GNNV) [48], zebrafish ISG15 against viral hemorrhagic septicemia virus [49], and ISG from half-smooth tongue sole in head kidney lymphocytes against megalocytivirus [50]. By targeting this key antiviral effector, ISG15, viruses have evolved varying capacities to antagonize host ISG15 during the host–virus evolutionary arms race, resulting in inconsistent antiviral efficacy of ISG15 across different fish species. For instance, although ISG15 from orange-spotted grouper can protect against GNNV infection, it fails to effectively reduce the pathogenicity of Singapore grouper iridovirus [51]. This study found that GCRV inhibits the anti-GCRV activity of ISG15 via NHRE3, suggesting that this mechanism may be a major contributor to the severe pathogenicity of GCRV in grass carp.

However, the antiviral mechanism of ISG15 in fish remains unclear. Human ISG15 can bind to viral or host proteins, directly blocking key steps in the viral life cycle or restoring host antiviral responses through ISGylation [21,52–54]. Studies have shown that fish ISG15 can bind to viral proteins. For example, zebrafish ISG15 interacts with P and N proteins of infectious hematopoietic necrosis virus [49], and Atlantic salmon (*Salmo salar*) ISG15 binds to infectious salmon anemia virus proteins [47]. However, in this study, we found no interaction between ISG15 and the GCRV NS1, NS3, or NS5 proteins (S4 Fig). Furthermore, human ISG15 has been reported to modify IRF3, preventing its degradation via ubiquitination, thereby enhancing IRF3 stability and maintaining it in an activated state [26,55]. Consistent with this, we found that grass carp ISG15 interacts with IRF3, an upstream regulator of IFN signaling, and promotes the expression of IRF3 downstream immune genes (*ifn1*, *ifn3*, *il6*, *il8*, *mx2*) (Fig 8). This represents one mechanism by which fish ISG15 exerts its antiviral function and highlights the conservation of innate immune regulatory pathways from lower to higher vertebrates.

In mammals, viral immune evasion strategies centered on ISG15 primarily involve inhibiting ISGylation [27,28] or inducing de-ISGylation [29,30], rather than the direct degradation of ISG15 observed in fish. A possible explanation is that fish possess ISG152, a protein highly homologous to ISG15 and differing by only three missense mutations (at positions 46, 48, and 50) and one synonymous mutation (at position 18) (S5 Fig). Furthermore, NHRE3 interacted with ISG152 (S5 Fig), and their functions were similar. Mammals lack such a functionally complementary gene for ISG15. Moreover, ISG15 is involved in additional cellular functions, such as maintaining genomic stability [56]. Direct degradation of the ISG15 protein would therefore be inefficient for the organism. Consequently, more refined and energy-efficient regulatory mechanisms, such as the inhibition of ISGylation and induction of deISGylation, may have evolved. In summary, investigating ubiquitination-mediated immune evasion by fish viruses provides insight into the evolutionary origins of infection immunology. This study identifies a fish virus–specific immune evasion pathway that negatively regulates the IFN response, further revealing the molecular dynamics of host–virus interactions. These findings provide continuous inspiration and potential targets for the development of effective and safe antiviral strategies.

## Materials and methods

### Viruses, cells, experimental fish, and challenge experiments

The GCRV strain was isolated and stored in our laboratory [57]. Grass carp ovary (GCO), *Ctenopharyngodon idella* kidney (CIK), and Human Embryonic Kidney 293T (HEK293T) cells were purchased from the China Center for Type Culture Collection. GCO and CIK cells were cultured in M199 medium supplemented with 10% fetal bovine serum, 100 U/mL penicillin, and 100 μg/mL streptomycin in a constant-temperature incubator at 28°C with 5% CO_2_. 293T cells were cultured in high-glucose DMEM supplemented with 10% fetal bovine serum, 100 U/mL penicillin, and 100 μg/mL streptomycin in a constant-temperature incubator at 37°C with 5% CO_2_. For viral challenge experiments, cells were seeded into 6-well plates. When the cells reached 80% confluence the following day, the medium was replaced with fresh complete medium containing GCRV (5 × 10 TCID_50_/mL) for infection, and the cells were harvested at designated time points.

Rare minnows used in this study were obtained from the National Aquatic Biological Resource Center of Institute of Hydrobiology, Chinese Academy of Sciences. The fish were reared in a recirculating water system under a 12-h light/dark cycle at approximately 25°C. Larvae were fed Artemia twice daily, and adult fish were fed red worms twice daily. Experimental fish were selected based on normal body shape, with all individuals being six months old from the same batch and of similar size, and acclimated in the experimental water prior to the experiments. Healthy fish were challenged using a high-salinity immersion method developed by our laboratory.

Grass carp used in this study were obtained from the Guanqiao Base of Institute of Hydrobiology, Chinese Academy of Sciences. Fish were cultured in outdoor ponds and fed duckweed and formulated feed. Experimental fish were selected based on normal body shape, with all individuals from the same batch and of similar size (approximately 10 cm). They were then transferred to indoor cement tanks for acclimatization before experimentation. Healthy fish were then challenged using the immersion method.

The rearing, sampling, and viral infection of experimental fish were conducted in accordance with the *Guide for the Care and Use of Laboratory Animals* (Ministry of Science and Technology of China, 2006), while all experimental procedures were approved by the Ethics Committee of the Institute of Hydrobiology, Chinese Academy of Sciences (E4552004).

### Plasmid construction and cell transfection

pcDNA-NHRE3-HA, pcDNA-NHRE3_N-HA, pcDNA-NHRE3_C-HA, pCMV-ISG15-MYC, and pcDNA-IRF3-HA were used for transient transfection and eukaryotic expression, while pEGFP-NHRE3, pEGFP-IRF3, and pmCherry-ISG15 were used to observe fluorescence colocalization. The vector backbone for the HA-tagged series was pcDNA3.1, with HindIII and EcoRI restriction sites. The vector backbone for pCMV-ISG15-MYC was pCMV-MYC, with BglII and NotI restriction sites. The vector backbones for pEGFP-NHRE3, pEGFP-IRF3, and pmCherry-ISG15 were pEGFP-N3 and pmCherry-N3, respectively, with XhoI and BamHI restriction sites.

pCDNA-Ub-his-K11O, pCDNA-Ub-his-K48, pCDNA-Ub-his-K63O, and pCDNA3.1-Ub-his-WT were used for ubiquitination experiments. In these constructs, all lysine (K) residues in the wild-type (WT) Ub sequence were replaced with arginine (R), while retaining lysine at positions 11 (K11O), 48 (K48O), and 63 (K63O), respectively, and adding a His tag at the N-terminus. The mutant ubiquitin sequences were synthesized by Wuhan Genecreate Biological Engineering Co., Ltd (Wuhan China). The vector backbone was pCDNA3.1-Ub-his-WT, with HindIII and EcoRI restriction sites. pCDNA3.1-Ub-his-WT was purchased from Wuhan Miaoling Biotechnology Co., Ltd (Wuhan China).

Homologous recombination between the digested vectors and insert fragments was performed using a homologous recombination enzyme (Vazyme, China) following the manufacturer's instructions. All restriction endonucleases were purchased from New England Biolabs, and primer sequences for homologous arms are listed in S3 Table.

For transfection, cells in the logarithmic growth phase were seeded into 6-well plates. When the cells reached 60%–80% confluence, recombinant plasmids were transfected using Lipofectamine 2000 transfection reagent (Invitrogen, USA), with the corresponding empty vector plasmids transfected as controls. Transfections were performed according to the manufacturer's instructions, and subsequent experiments were conducted 24 h post-transfection, depending on the experimental objectives.

### Construction of rare minnow mutant strains

The target sequence for rare minnow *nhre3* gene knockout (GGTGGCAGTAGCCACAGTAC) was designed using CRISPOR version 5.01 (CRISPOR; tefor.net). Using the pT7-gRNA plasmid as a template, PCR primers were designed to amplify a fragment containing the T7 promoter, the target gene site, and the gRNA sequence. After sequence verification, RNA was synthesized using the MEGAshortscript T7 Transcription Kit (Invitrogen, USA) and purified using the mirVana miRNA Isolation Kit (Invitrogen, USA). RNA concentration was determined, and samples were stored at −80°C.

For microinjection into rare minnow embryos, the gRNA targeting the *nhre3* site was mixed with Cas9 protein (Invitrogen, USA) and diluted to a final concentration of 300 ng/μL for gRNA and 100 ng/μL for Cas9. The mixture was injected into one-cell-stage rare minnow embryos using the MPPI-3 microinjection system (Applied Scientific Instrumentation, USA), and the injected embryos were subsequently cultured at 25°C. After 24 h, 50 embryos were randomly selected, and genomic DNA was extracted from each embryo using the Universal Genomic DNA Kit (CWBIO, China). PCR amplification was then performed using the target detection primers KONHRE3F/R, followed by sequencing to verify and assess mutation efficiency in the P0 generation. Primer sequences are listed in S3 Table.

After challenge of the P0 generation, surviving individuals were crossed with wild-type fish to obtain the F1 generation. The F1 generation was then intercrossed to generate homozygous F2 mutants. The surviving P0 individuals, as well as the F1 and F2 generations, were genotyped using the target detection primers.

### RNA extraction, reverse transcription, and qPCR

Total RNA was extracted from all samples using TRIzol reagent (Invitrogen, USA), and reverse transcription was performed using the HiScript II 1st Strand cDNA Synthesis Kit (Vazyme, China). qPCR reaction mixtures were prepared using the HiScript II Q RT SuperMix for qPCR (+gDNA wiper) (Vazyme, China). qPCR was performed on a CFX96 Real-Time PCR Detection System (Bio-Rad). Each sample was run in triplicate, with the β-actin gene used as an internal reference to normalize gene expression. The annealing/extension temperature (T_m_) for amplification was 60°C. Relative expression levels of GCRV segments and genes were calculated using the 2^−∆∆Ct^ method. All qPCR primers are listed in S4 Table.

### Protein extraction, western blotting, and antibody preparation

To lyse the cells, the culture medium was aspirated, and the cells were washed twice with ice-cold Phosphate Buffered Saline (PBS). Then, 500 μL of Western and IP lysis buffer (Beyotime, China) containing 1% Phenylmethanesulfonyl fluoride (PMSF) was added, and the cells were lysed on a horizontal shaker in a 4°C refrigerator for 30 min. The lysate from the culture dish was collected, transferred to a microcentrifuge tube, and centrifuged at 13,000 × g for 15 min at 4°C. The supernatant was then transferred to a new tube. After adding 5 × loading buffer and mixing, the samples were heated in boiling water for 5–10 min and then cooled on ice for subsequent western blotting.

The protein lysates were separated on 10% SDS-PAGE gels and then transferred to polyvinylidene fluoride membranes (Bio-Rad). The membranes were blocked at room temperature for 1 h with Tris Buffered Saline with Tween (TBST) buffer containing 5% skim milk, followed by 16-18h incubation at 4°C with appropriately diluted specific primary antibodies. After three washes with TBST, the membranes were incubated with secondary antibodies for 2 h at room temperature. Following another three washes with TBST buffer, the protein bands were detected using the Immobilon Western Chemiluminescent HRP Substrate (Millipore) and visualized with the ImageQuant LAS 4000 System (GE Healthcare).

Rabbit anti-HA tag, rabbit anti-MYC tag, rabbit anti-β-actin, mouse secondary, and rabbit secondary antibodies were purchased from Wuhan ABclonal Biotechnology Co., Ltd. Mouse anti-ISG15 antibody was purchased from Santa Cruz Biotechnology. The brief procedure for preparing anti-NHRE3 antibodies is as follows: NHRE3 protein (611–741aa) with a 6 × His tag was expressed in *Escherichia coli* and purified using a nickel affinity column. The purified NHRE3 protein was used to immunize rabbits via four injections, with intervals of 28, 14, and 14 d. The first injection was administered with Freund's Complete Adjuvant, while subsequent injections were administered with Freund's Incomplete Adjuvant. Three days after the last injection, antiserum titer was determined via ELISA. The rabbit exhibiting the highest antibody titer was euthanized, and serum was collected on day 64 after the initial injection. An affinity chromatography column was prepared by coupling 1 mg of purified NHRE3 protein to CNBr-activated Sepharose 4B resin (GE Healthcare). The antiserum was passed through the column, and specific antibodies were eluted with glycine–hydrochloric acid buffer (pH 2.5). Purified antibodies were obtained after dialysis and concentration. SDS-PAGE analysis of the purified NHRE3 antigen protein is shown in S6 Fig.

### Immunoprecipitation–Mass Spectrometry (IP–MS) and Co-Immunoprecipitation (Co-IP)

GCO cells were transfected with pCDNA-HA-CiNHRE3. After 24 h, the cells were infected with GCRV, and samples were collected 24 h post-infection. A mock-infected group (transfected with pCDNA-HA-CiNHRE3 but not infected with GCRV) was also included. Cellular proteins were extracted and immunoprecipitated using HA magnetic beads (Thermo Fisher Scientific, USA) overnight at 4°C, following the manufacturer's instructions. An aliquot of the immunoprecipitated sample was analyzed using SDS-PAGE to verify IP efficiency. The remaining beads were sent to Wuhan Pudu Zhonghe Biotechnology Co., Ltd. for MS analysis. The raw IP-MS data has been uploaded to the Integrated Proteome Resources (iProX) database under project ID IPX0017660000.

For Co-IP experiments, 293T cells were transfected with pCDNA-NHRE3-HA, pCDNA-NHRE3_N-HA, pCDNA-NHRE3_C-HA, PCMV-ISG15-MYC, or pCDNA-IRF3-HA according to experimental objectives. After 24 h, the culture medium was discarded, residual medium was removed by washing with ice-cold PBS, and the cells were lysed using lysis buffer. Following centrifugation at 13,000 rpm, 50 μL of the lysate was saved as the input sample. The remaining lysate was incubated with HA magnetic beads overnight at 4°C for Co-IP, following the manufacturer's protocol. Finally, 50 μL of 1 × loading buffer was added to the washed beads, and these samples, along with the previously collected input samples, were analyzed via SDS-PAGE.

### Intracellular ubiquitination assay

GCO cells were transfected with pcDNA-HA-NHRE3, pCMV-MYC-ISG15, or pCMV-Ub-His plasmids (wild-type and various ubiquitin mutants) according to experimental objectives. A control group without pcDNA-HA-NHRE3 transfection was also included. After 18 h, the cells were treated with MG132 for 6 h. At 24 h post-transfection, the culture medium was discarded, residual medium was removed by washing with ice-cold PBS, and the cells were lysed using lysis buffer. Following centrifugation at 13,000 rpm, 50 μL of the lysate was saved as the whole-cell lysate (WCL) sample. The remaining lysate was incubated overnight at 4°C with magnetic beads conjugated with His-tag antibody (MedChemExpress, USA), following the manufacturer's instructions. Finally, 50 μL of 1 × loading buffer was added to the washed beads, and these samples, along with the previously collected WCL samples, were analyzed using SDS-PAGE.

### Intracellular colocalization assay

GCO cells in the logarithmic growth phase were seeded into glass-bottom culture dishes (Sorfa, China). When the cells reached approximately 80% confluence, the recombinant plasmids pEGFP-NHRE3 and pmCherry-ISG15 were co-transfected into the cells. At 24 h post-transfection, the culture medium was removed, and the cells were washed three times with PBS. The cells were then fixed with 4% paraformaldehyde, washed three additional times with PBS, and stained with DAPI (Beyotime, China). The localization of fluorescent signals was observed using a Leica live-cell scanning confocal microscope. Colocalization was quantified using the Colocalization Finder plugin in ImageJ [58]. The Pearson correlation coefficient, ranging from −1–1, was interpreted as follows: values between −1 and −0.5 indicated no colocalization, whereas values between 0.5 and 1 indicated colocalization.

### Statistical analysis

Survival curves were calculated using the Kaplan–Meier method and analyzed with the log-rank test in GraphPad Prism 9.3.1 software (GraphPad Inc., San Diego, CA, USA). Other statistical analyses were performed using an independent-samples *t*-test with SPSS software (version 21.0; IBM, New York, NY, USA). All data were derived from at least three independent replicate experiments, and error bars represent the mean ± standard deviation. A *P*-value < 0.05 was considered statistically significant, with significance denoted as follows: ns (not significant), * *P* < 0.05, ** *P* < 0.01, and *** *P* < 0.001.

## Supporting information

S1 Fig The NHRE3 HECT domain possesses a conserved E2 ubiquitin ligase binding site and an E3 ubiquitin ligase catalytic site. (A) The NHRE3 HECT domain possesses a conserved E2 ubiquitin ligase binding site, with yellow regions indicating conserved residues. (B) The NHRE3 HECT domain possesses a conserved E3 ubiquitin ligase catalytic site, with yellow regions indicating conserved residues.
(DOCX)

S2 Fig Mutations in the *nhre3* do not affect the growth and development of *Gobiocypris rarus.* (A) Comparison of wild-type and mutant *Gobiocypris rarus* larvae at 4 days post-hatch (dph) showed no significant differences. (B) Comparison of wild-type and mutant *Gobiocypris rarus* adult fish (approximately 5 months old) showed no significant differences. (C) Comparison of body length between wild-type and mutant *Gobiocypris rarus* adults (n = 15). No significant difference was observed. (D) Comparison of body weight between wild-type and mutant Gobiocypris rarus adults (n = 15). No significant difference was observed.
(DOCX)

S3 Fig Tissue-specific expression of *nhre3* in *Gobiocypris rarus* and *Ctenopharyngodon Idella.* (A) Tissue-specific expression of *nhre3* in *Gobiocypris rarus* detected by qPCR. (B) Tissue-specific expression of *nhre3* in *Ctenopharyngodon Idella* detected by qPCR.
(DOCX)

S4 Fig ISG15 does not interact with GCRV NS1, NS3, and NS5. (A) Co-expression of ISG15 and NS1 in GCO cells, followed by co-immunoprecipitation (COIP) analysis for interaction detection after 24 hours. (B) Co-expression of ISG15 and NS3 in GCO cells, followed by COIP analysis for interaction detection after 24 hours. (C) Co-expression of ISG15 and NS5 in GCO cells, followed by COIP analysis for interaction detection after 24 hours.
(DOCX)

S5 Fig ISG15 and ISG152 are highly homologous. (A) A comparison of the amino acid sequences of ISG15 and ISG152 reveals only three missense mutations (at positions 46aa, 48aa, and 50aa) and one synonymous mutation (at position 18aa). (B) COIP performed 24 hours after co-transfection of pcDNA-NHRE3-HA and pcDNA-ISG152-MYC in 293T cells revealed distinct bands in the NHRE3 and ISG152 IP fractions.
(DOCX)

S6 Fig Purification of the NHRE3 antigen protein. M: marker, IN: Input, FT: Flow Through, W: Wash, E: Elution.
(DOCX)

S1 Table Candidate interacting proteins of NHRE3 in the control group. (DOCX)

S2 Table Candidate interacting proteins of NHRE3 in the experimental group. (DOCX)

S3 Table Knockout verification and homologous arm primers. (DOCX)

S4 Table qPCR primers. (DOCX)

S1 Raw Gel Original images for blots and gels in article’s figures and supporting information files. (PDF)
