# Supplementary material for: A fish-specific E3 ubiquitin ligase NHRE3 suppresses antiviral immunity by degrading ISG15 via ubiquitination
Source: PLoS Pathog. 2026 Sep 29;22(9):e1014544. doi: 10.1371/journal.ppat.1014544 (PMC13623101; doi:10.1371/journal.ppat.1014544)
Supplement: Supplementary file 1 [file ppat.1014544.s011.pdf]

loading order: from left to right

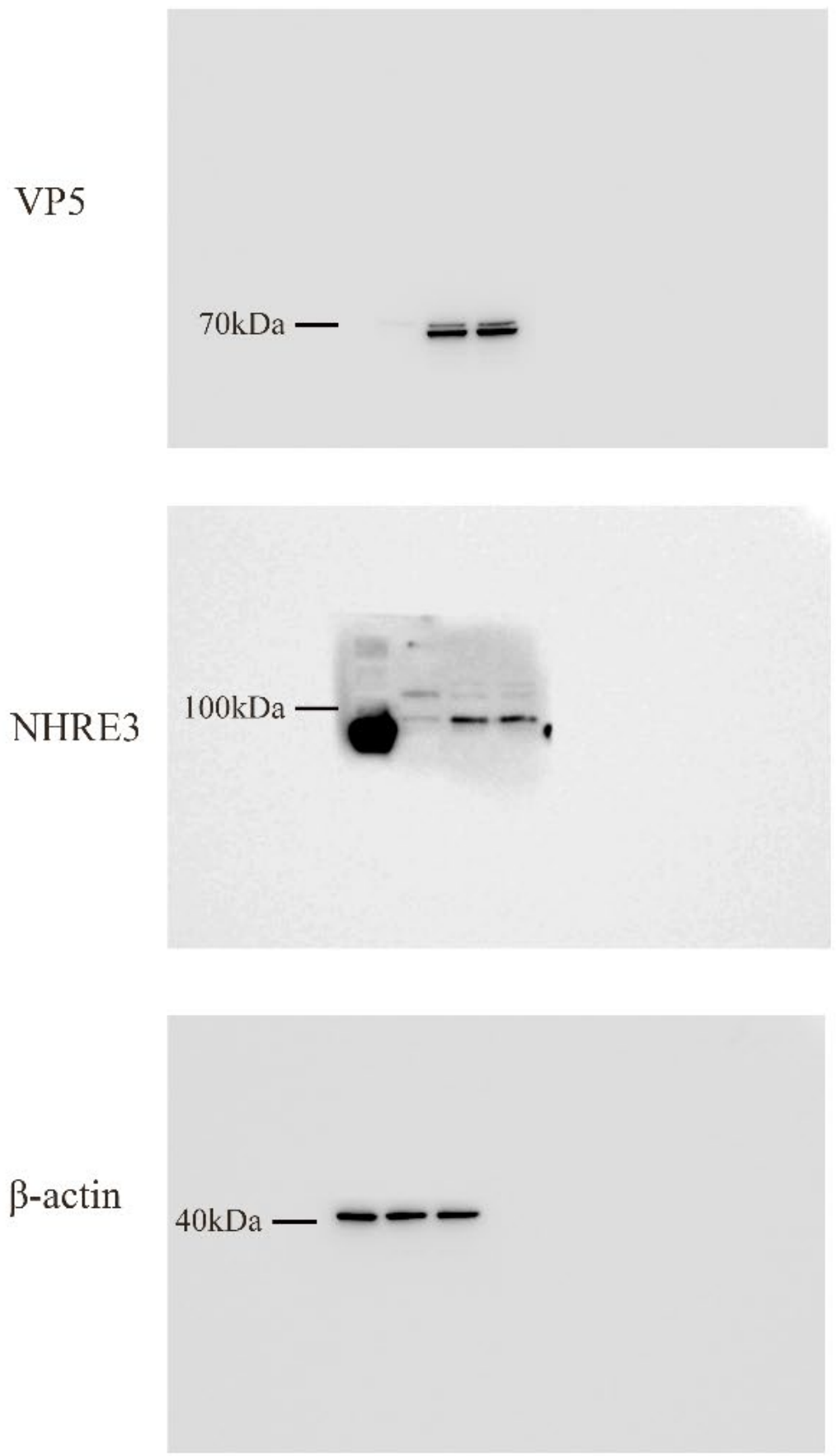

Fig3 D

loading order: from left to right

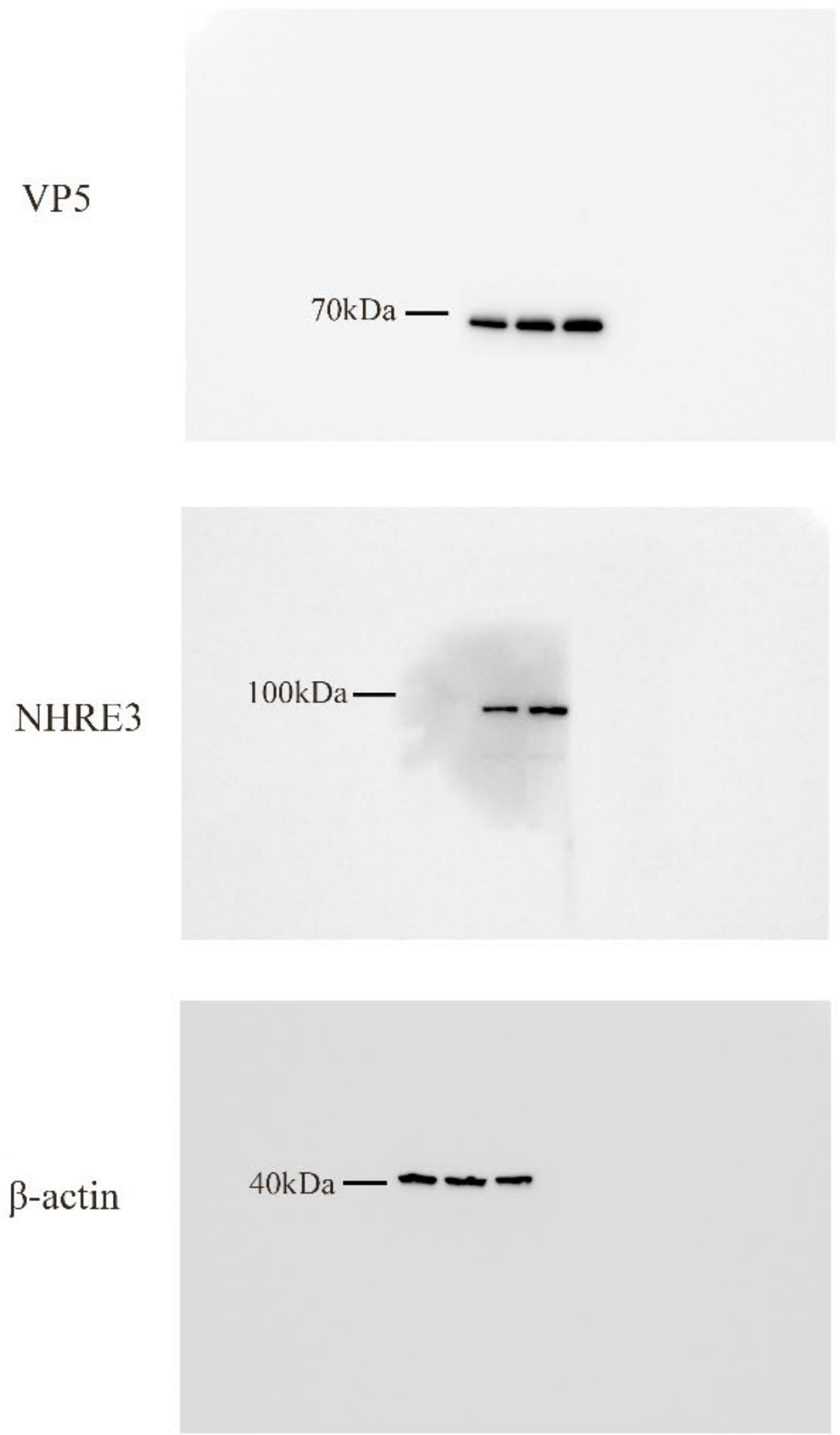

Fig3 H

exposure time: VP5 4s, NHRE3 60s, β-actin 1/2s

loading order: from left to right

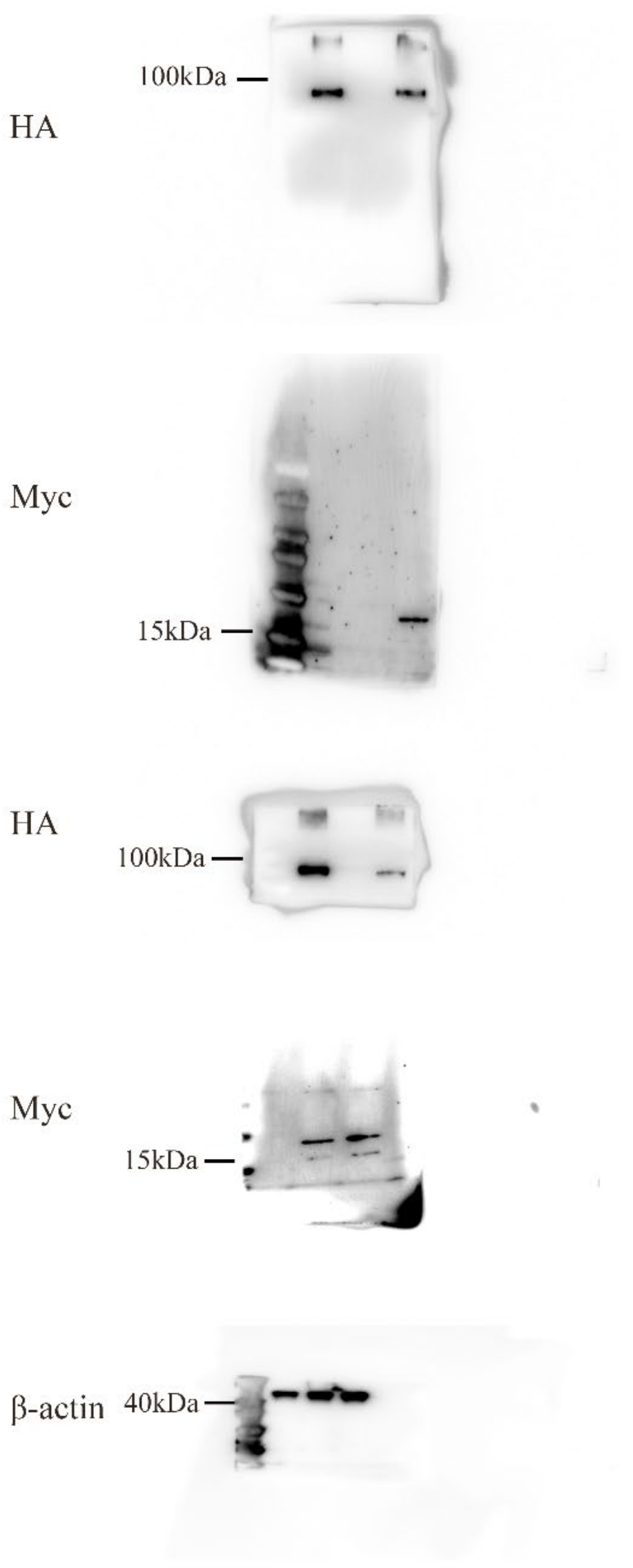

Fig4 E

exposure time: HA 60s, Myc 30s, β-actin 1s

loading order: from left to right

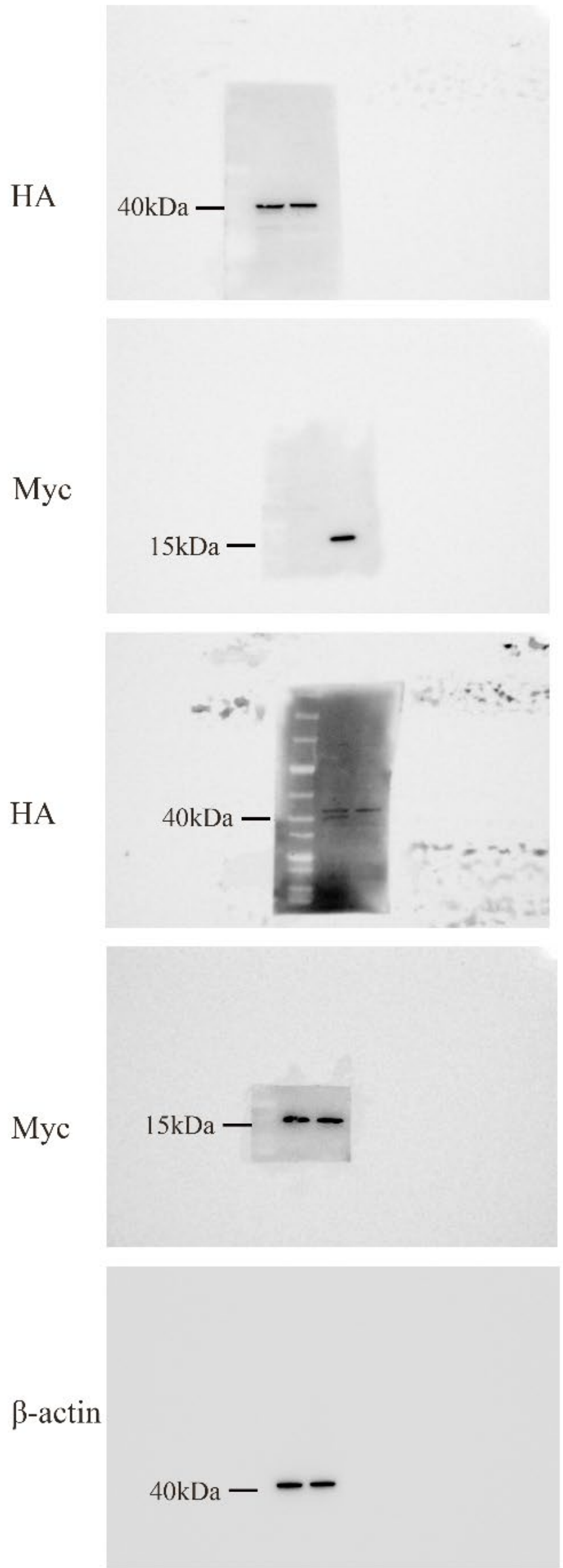

Fig4 H

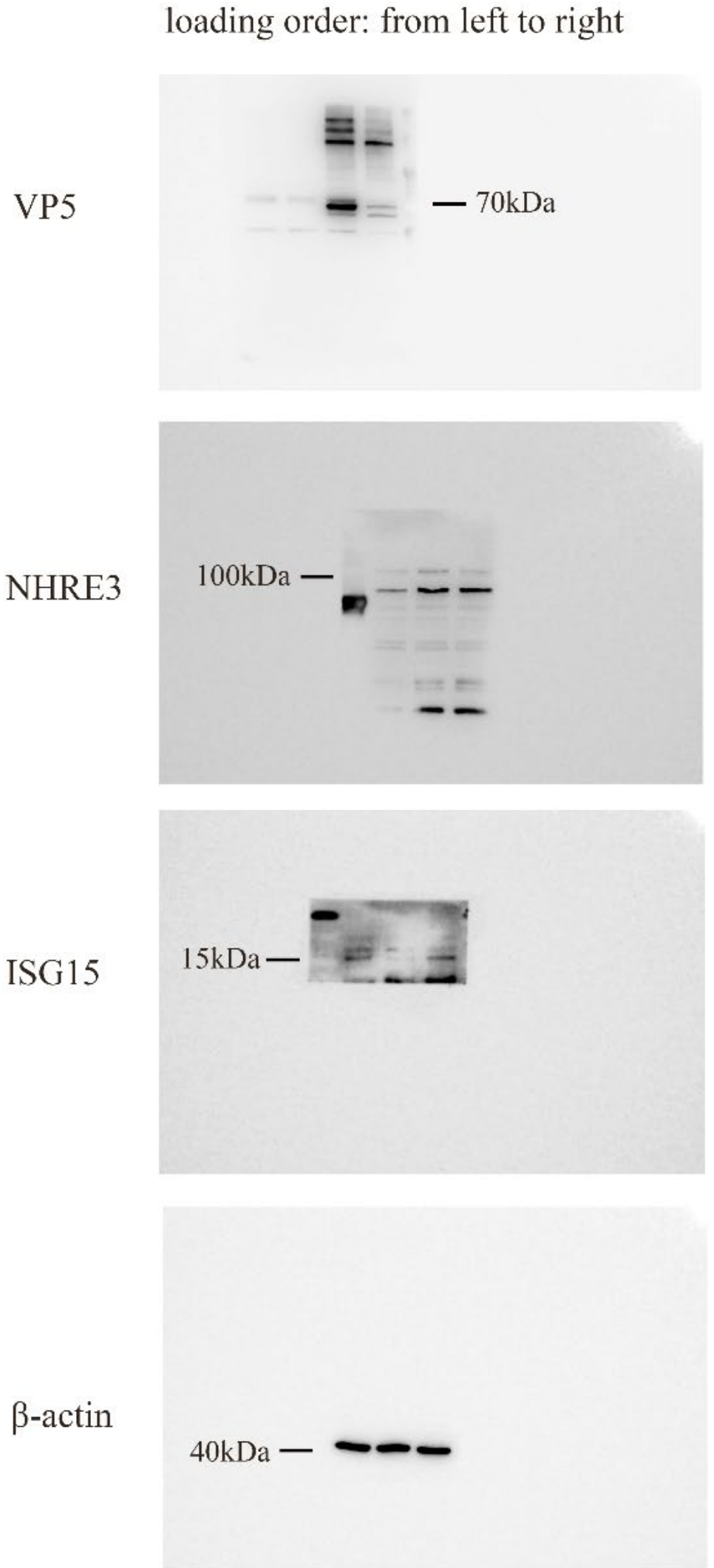

Fig5 A

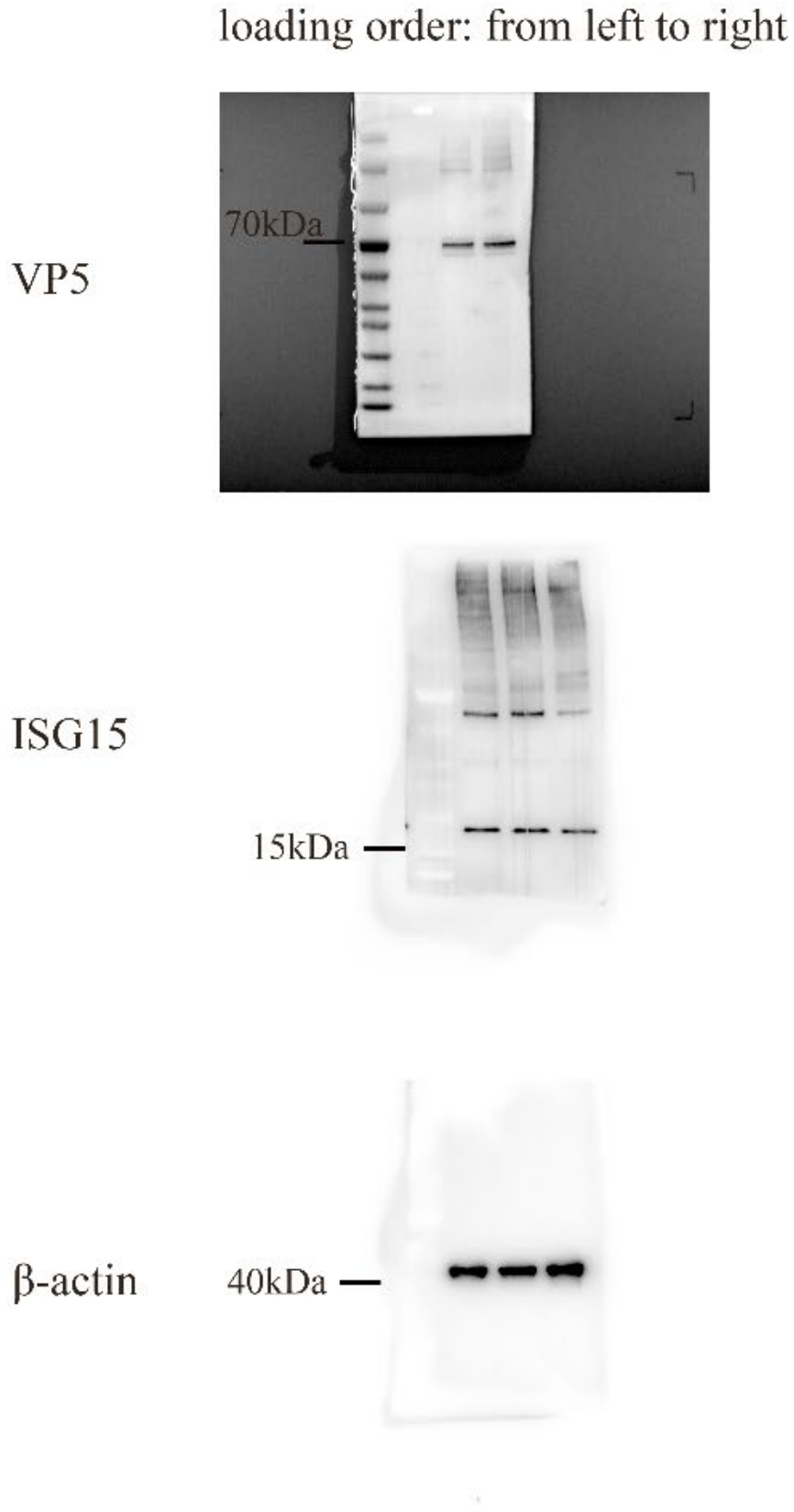

Fig5 B

exposure time: VP5 4s, NHRE3 60s, ISG15 60s, β-actin 1s

loading order: from left to right

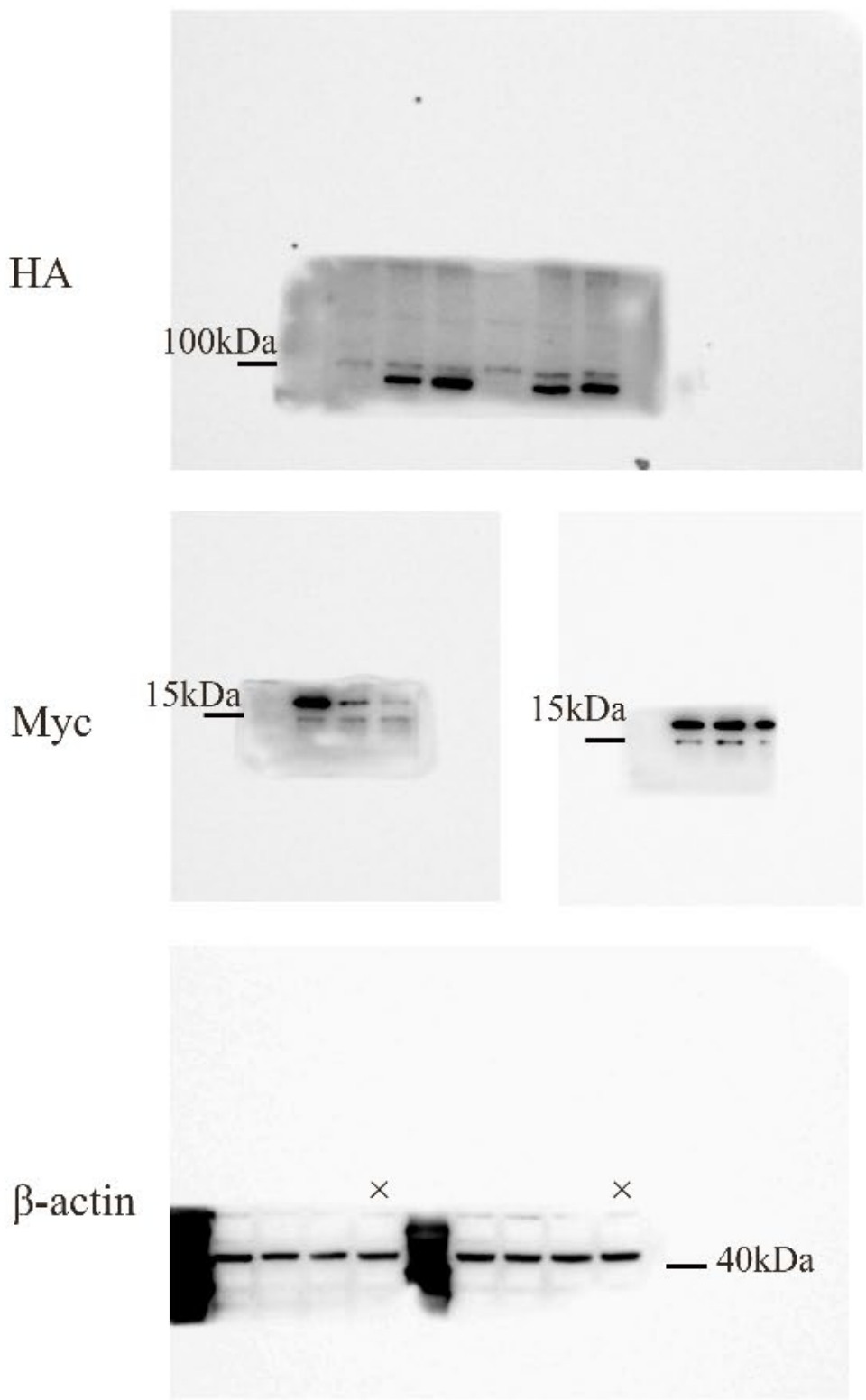

Fig5 D

loading order: from left to right

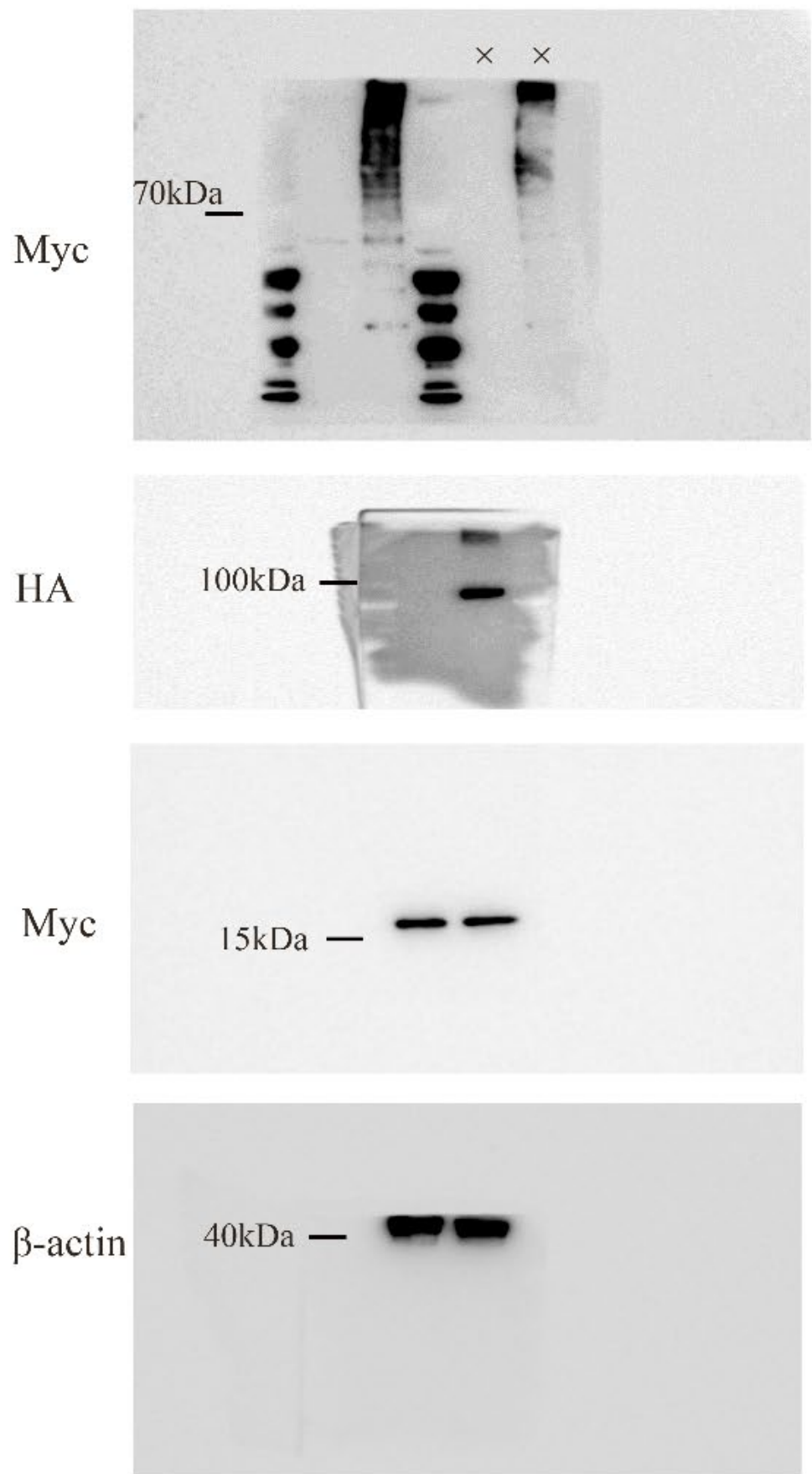

Fig5 E

exposure time: HA 60s, Myc 30s, β-actin 1s

loading order: from left to right

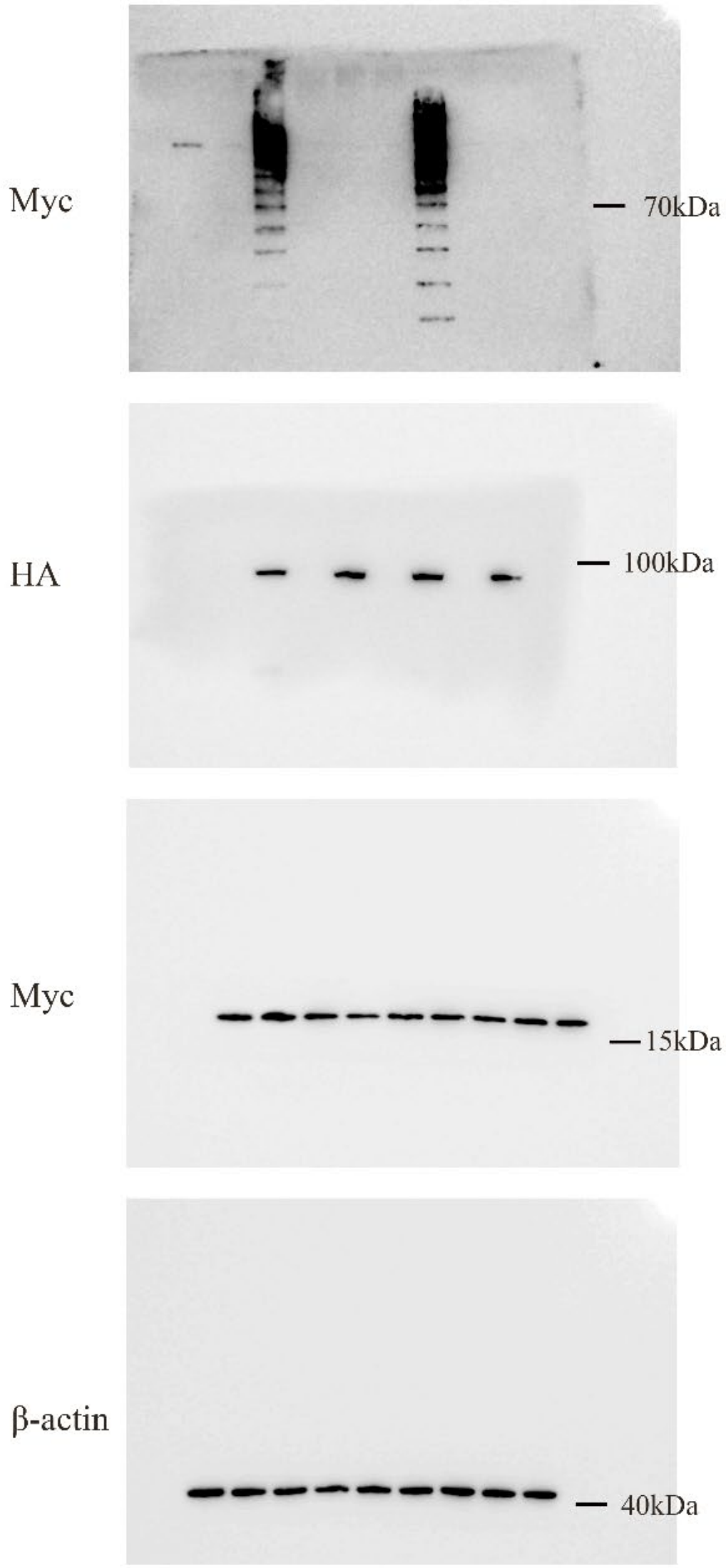

Fig5 F

exposure time: HA 60s, Myc 30s, β-actin 1s

loading order: from left to right

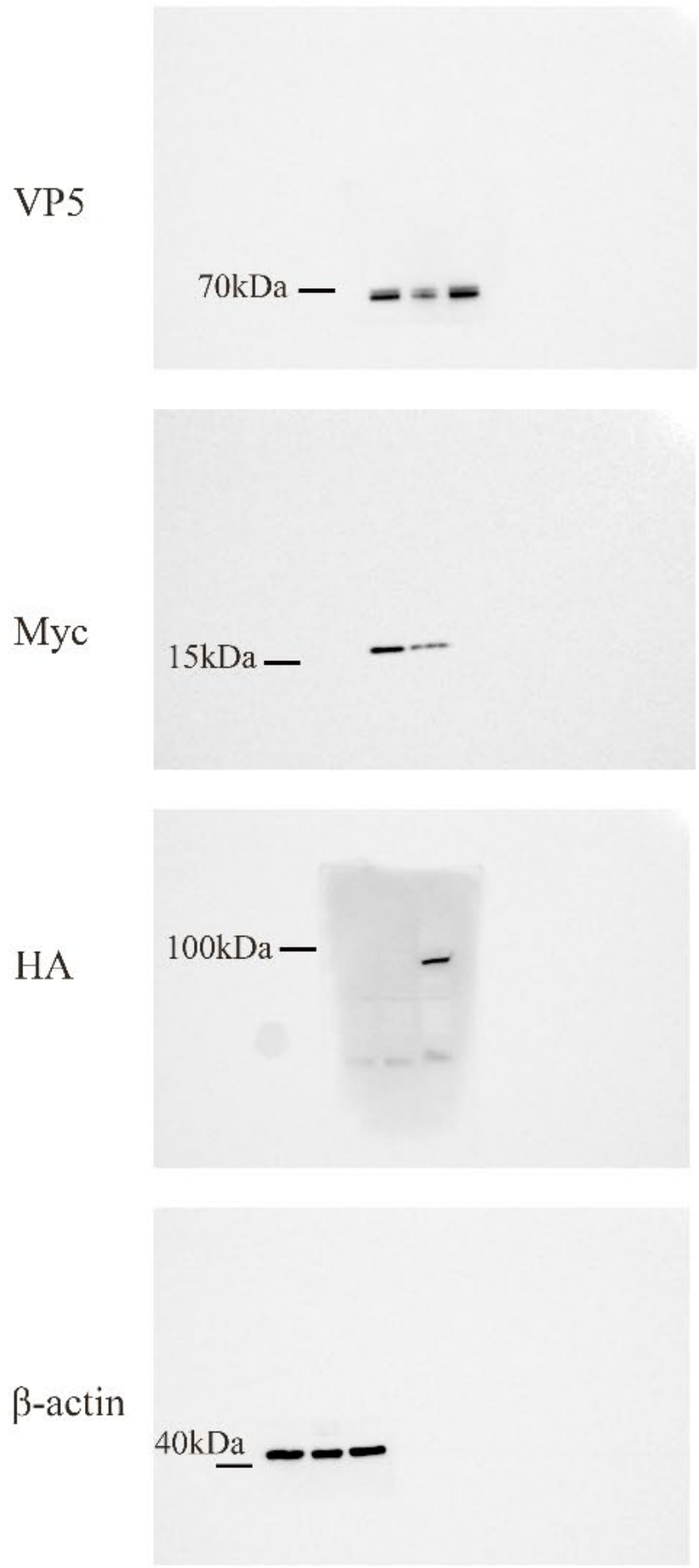

Fig6 B

loading order: from left to right

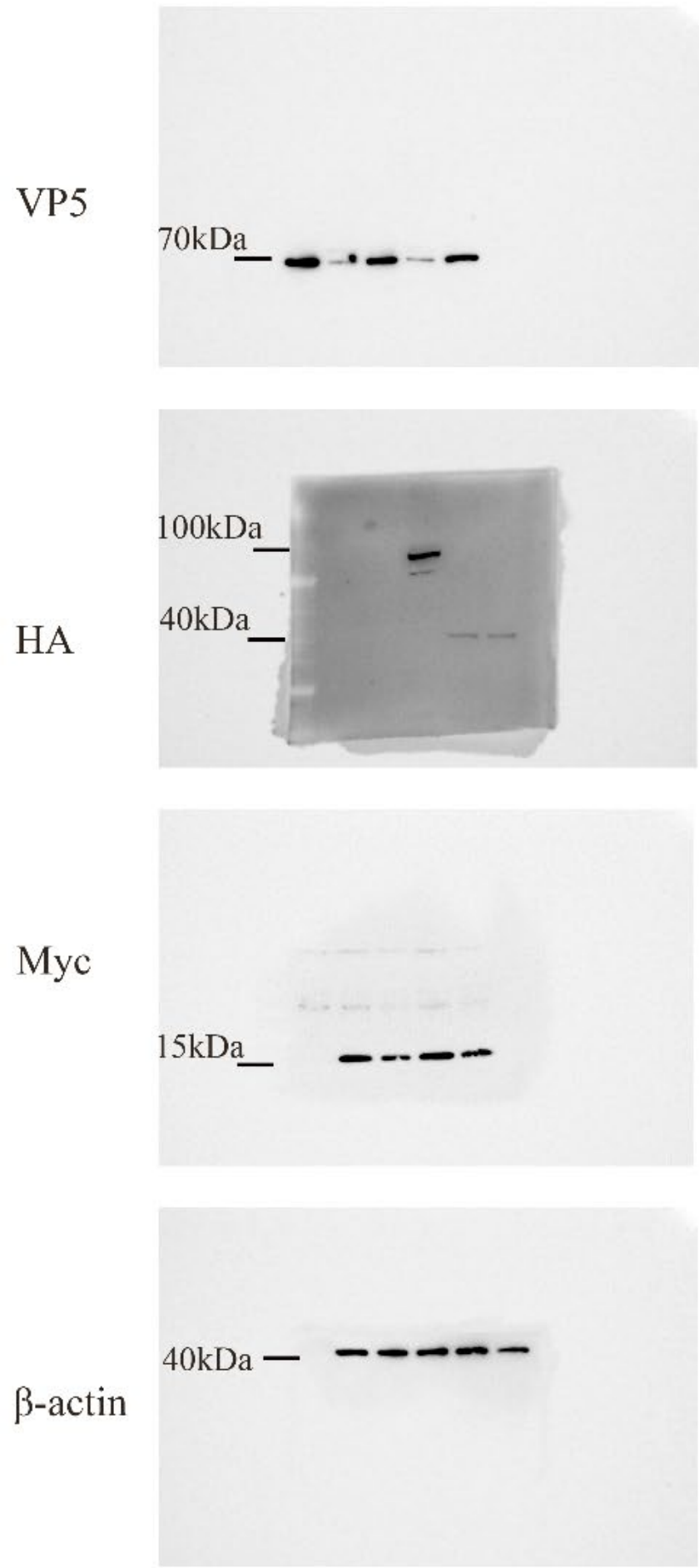

Fig6 E

exposure time: VP5 4s, HA 60s, Myc 30s, β-actin 1s

loading order: from left to right

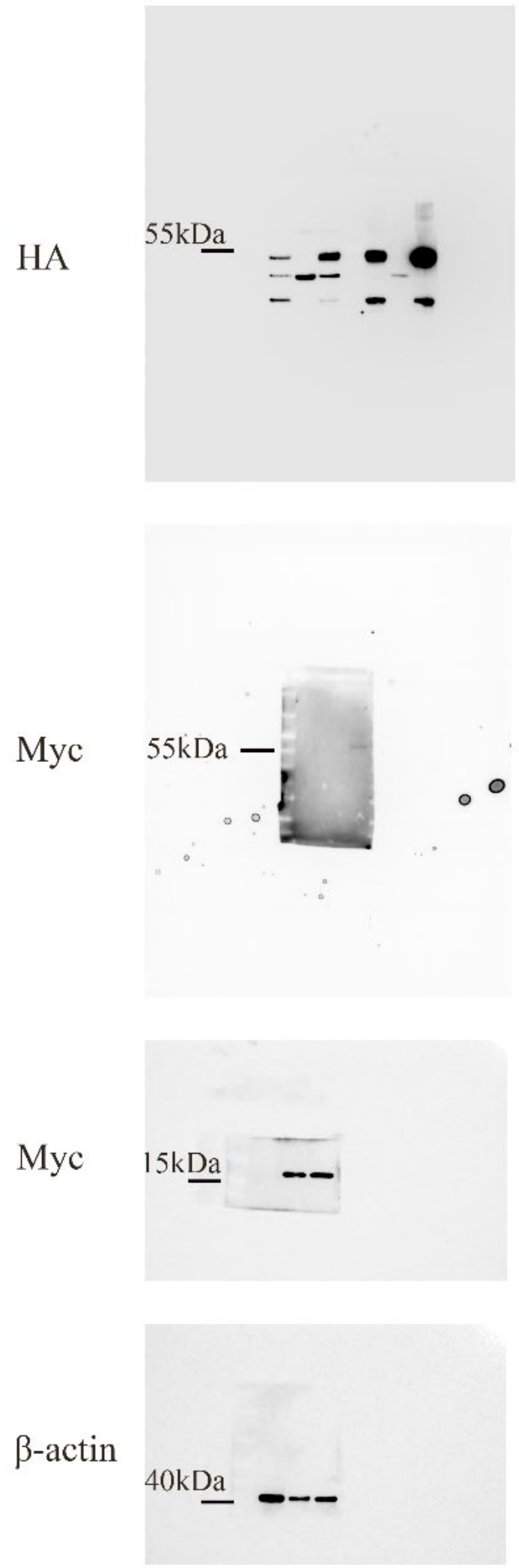

Fig7 A

exposure time: HA 30s, Myc 60s, β-actin 1s

loading order: from left to right

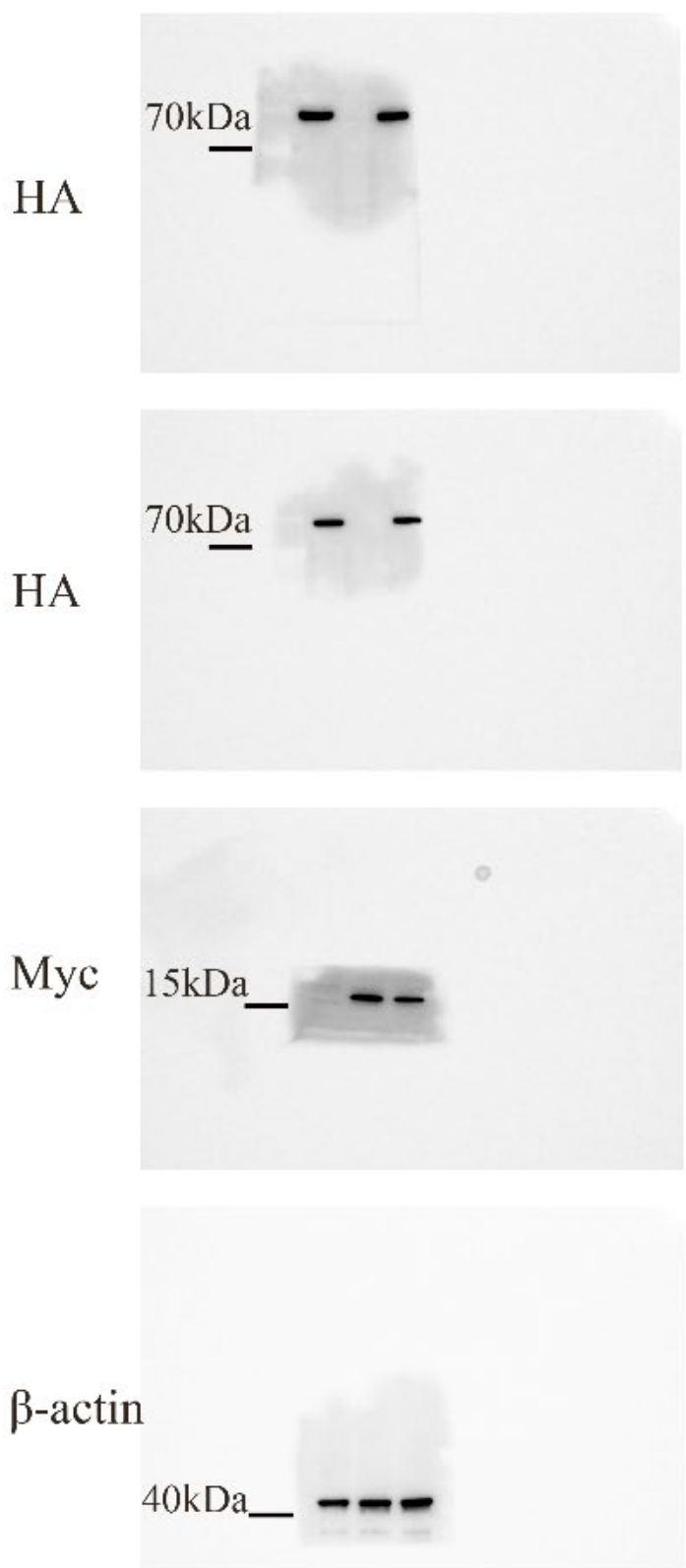

S4 Fig A

loading order: from left to right

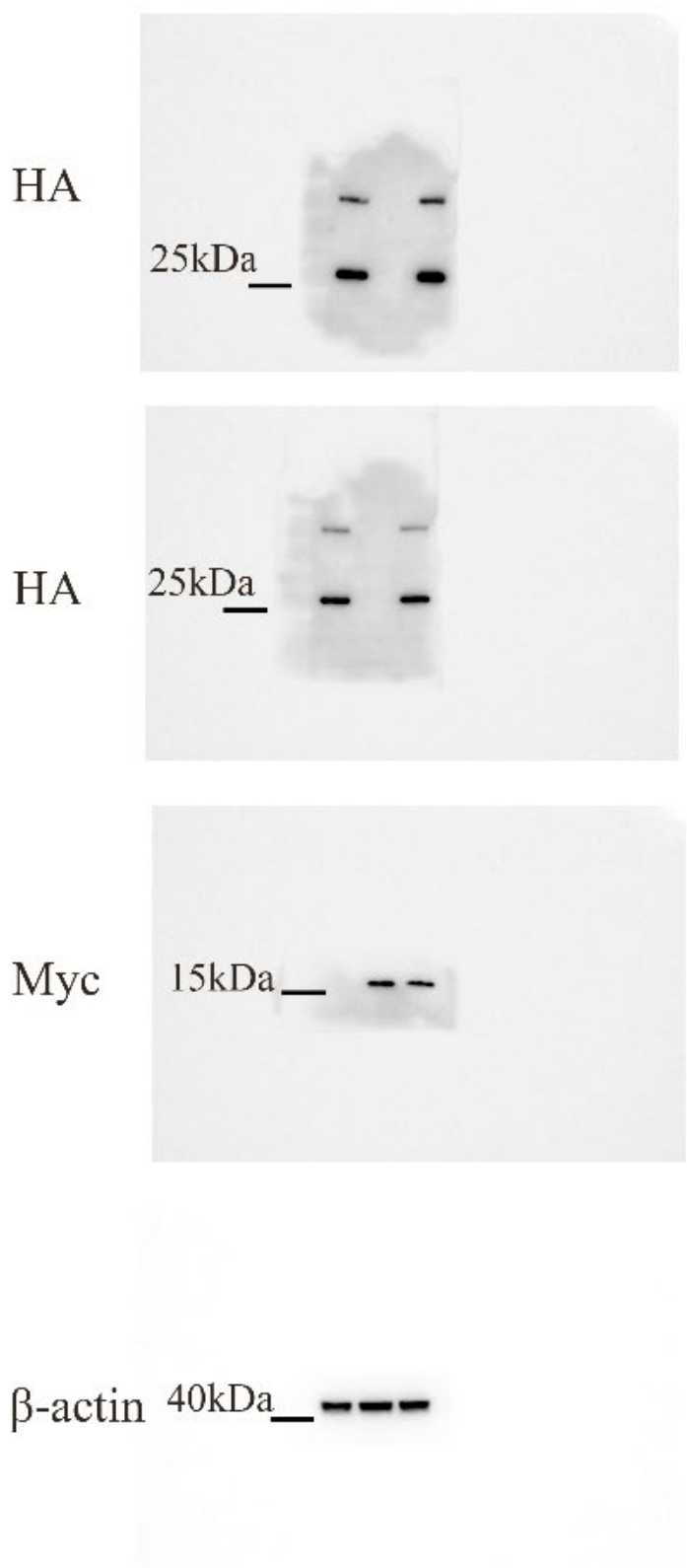

S4 Fig B

loading order: from left to right

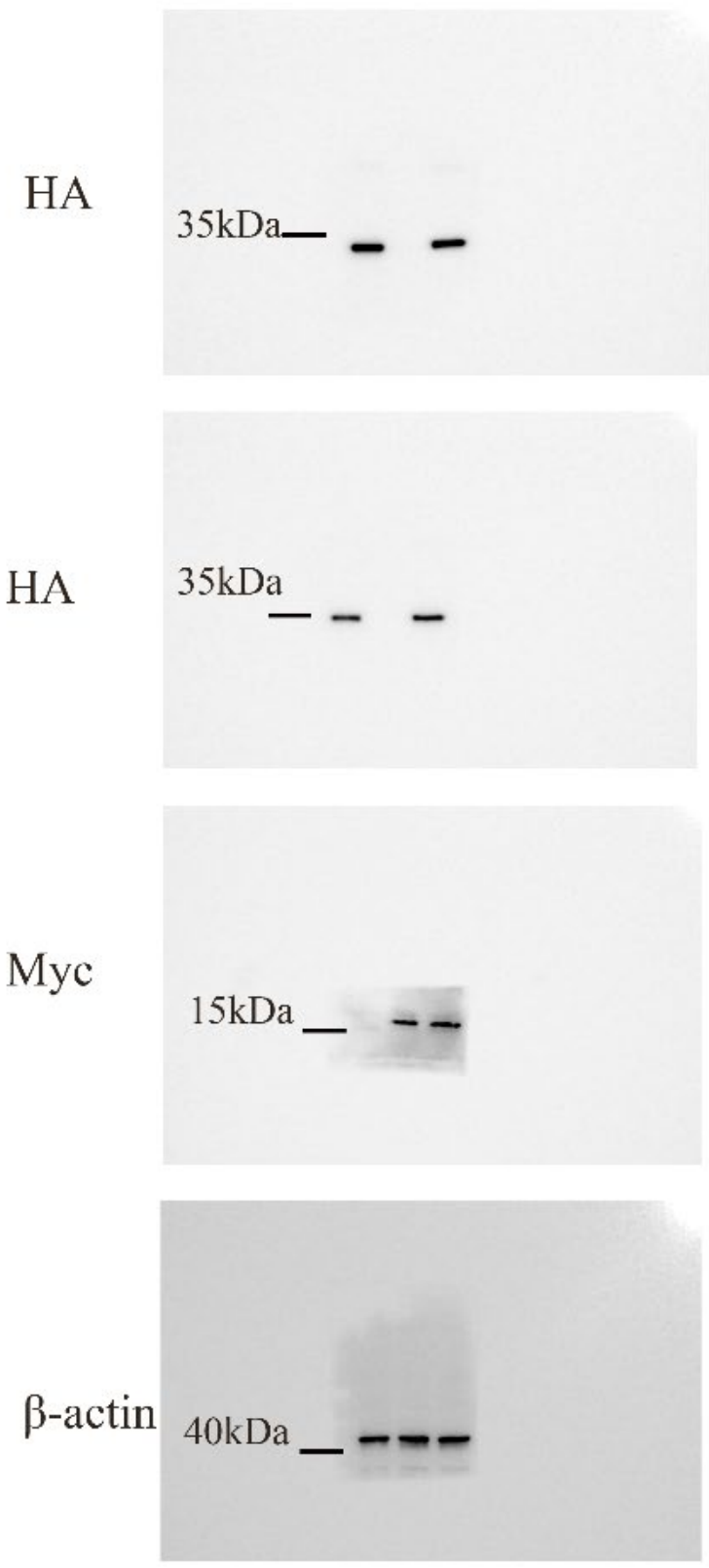

S4 Fig C

exposure time: HA 30s, Myc 30s, β-actin 1s

loading order: from left to right

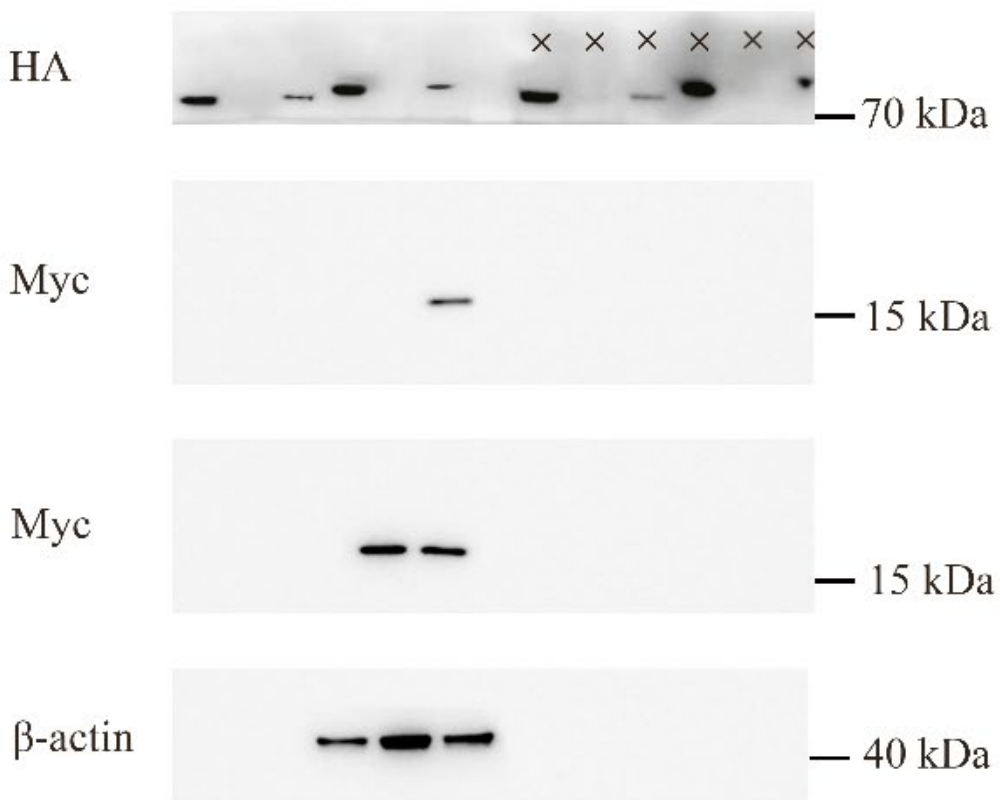

S5 Fig B

exposure time: HA 60s, Myc 30s, β-actin 1s
