# Supplementary material for: A fish-specific E3 ubiquitin ligase NHRE3 suppresses antiviral immunity by degrading ISG15 via ubiquitination
Source: PLoS Pathog. 2026 Sep 29;22(9):e1014544. doi: 10.1371/journal.ppat.1014544 (PMC13623101; doi:10.1371/journal.ppat.1014544)
Supplement: Supplementary file 2 [file ppat.1014544.s001.docx]

A

B

**S1 Fig.** **The NHRE3 HECT domain possesses a conserved E2 ubiquitin ligase binding site and an E3 ubiquitin ligase catalytic site.** (A) The NHRE3 HECT domain possesses a conserved E2 ubiquitin ligase binding site, with yellow regions indicating conserved residues. (B) The NHRE3 HECT domain possesses a conserved E3 ubiquitin ligase catalytic site, with yellow regions indicating conserved residues.
