# Supplementary material for: A fish-specific E3 ubiquitin ligase NHRE3 suppresses antiviral immunity by degrading ISG15 via ubiquitination
Source: PLoS Pathog. 2026 Sep 29;22(9):e1014544. doi: 10.1371/journal.ppat.1014544 (PMC13623101; doi:10.1371/journal.ppat.1014544)
Supplement: Supplementary file 3 [file ppat.1014544.s002.docx]

**S2 Fig.** **Mutations in the *nhre3* do not affect the growth and development of *Gobiocypris rarus*.** (A) Comparison of wild-type and mutant *Gobiocypris rarus* larvae at 4 days post-hatch (dph) showed no significant differences. (B) Comparison of wild-type and mutant *Gobiocypris rarus* adult fish (approximately 5 months old) showed no significant differences. (C) Comparison of body length between wild-type and mutant *Gobiocypris rarus* adults (n=15). No significant difference was observed. (D) Comparison of body weight between wild-type and mutant Gobiocypris rarus adults (n=15). No significant difference was observed.
