# Supplementary material for: A fish-specific E3 ubiquitin ligase NHRE3 suppresses antiviral immunity by degrading ISG15 via ubiquitination
Source: PLoS Pathog. 2026 Sep 29;22(9):e1014544. doi: 10.1371/journal.ppat.1014544 (PMC13623101; doi:10.1371/journal.ppat.1014544)
Supplement: Supplementary file 4 [file ppat.1014544.s003.docx]

**S3 Fig.** **Tissue-specific expression of *nhre3* in *Gobiocypris rarus* and *Ctenopharyngodon Idella*.** (A) Tissue-specific expression of *nhre3* in *Gobiocypris rarus* detected by qPCR. (B) Tissue-specific expression of *nhre3* in *Ctenopharyngodon Idella* detected by qPCR.
