# Supplementary material for: A fish-specific E3 ubiquitin ligase NHRE3 suppresses antiviral immunity by degrading ISG15 via ubiquitination
Source: PLoS Pathog. 2026 Sep 29;22(9):e1014544. doi: 10.1371/journal.ppat.1014544 (PMC13623101; doi:10.1371/journal.ppat.1014544)
Supplement: Supplementary file 5 [file ppat.1014544.s004.docx]

**S4 Fig.** **ISG15 does not interact with GCRV NS1, NS3, and NS5.** (A) Co-expression of ISG15 and NS1 in GCO cells, followed by co-immunoprecipitation (COIP) analysis for interaction detection after 24 hours. (B) Co-expression of ISG15 and NS3 in GCO cells, followed by COIP analysis for interaction detection after 24 hours. (C) Co-expression of ISG15 and NS5 in GCO cells, followed by COIP analysis for interaction detection after 24 hours.
