# Supplementary material for: A fish-specific E3 ubiquitin ligase NHRE3 suppresses antiviral immunity by degrading ISG15 via ubiquitination
Source: PLoS Pathog. 2026 Sep 29;22(9):e1014544. doi: 10.1371/journal.ppat.1014544 (PMC13623101; doi:10.1371/journal.ppat.1014544)
Supplement: Supplementary file 6 [file ppat.1014544.s005.docx]

**S5 Fig.** **ISG15 and ISG152 are highly homologous.** (A) A comparison of the amino acid sequences of ISG15 and ISG152 reveals only three missense mutations (at positions 46aa, 48aa, and 50aa) and one synonymous mutation (at position 18aa). (B) COIP performed 24 hours after co-transfection of pcDNA-NHRE3-HA and pcDNA-ISG152-MYC in 293T cells revealed distinct bands in the NHRE3 and ISG152 IP fractions.
