# Supplementary material for: A fish-specific E3 ubiquitin ligase NHRE3 suppresses antiviral immunity by degrading ISG15 via ubiquitination
Source: PLoS Pathog. 2026 Sep 29;22(9):e1014544. doi: 10.1371/journal.ppat.1014544 (PMC13623101; doi:10.1371/journal.ppat.1014544)
Supplement: Supplementary file 7 [file ppat.1014544.s006.docx]

NHRE3 antigen (611-741aa，35.5 kDa)

**S6 Fig. Purification of the NHRE3 antigen protein.** M: marker, IN: Input, FT: Flow Through, W: Wash, E: Elution.
