# Supplementary material for: A fish-specific E3 ubiquitin ligase NHRE3 suppresses antiviral immunity by degrading ISG15 via ubiquitination
Source: PLoS Pathog. 2026 Sep 29;22(9):e1014544. doi: 10.1371/journal.ppat.1014544 (PMC13623101; doi:10.1371/journal.ppat.1014544)
Supplement: Supplementary file 8 [file ppat.1014544.s007.docx]

**S1 Table. Candidate interacting proteins of NHRE3 in the control group**

| **Accession** | **Description** | **log2 intensity** | **log2 FC** |
| --- | --- | --- | --- |
| B3GN92 | Cellular nucleic acid-binding protein | 28.500398741 | 9.68 |
| A0A068L824 | Histone H2A (Fragment) | 29.274332188 | 9.16 |
| A0A7G8KPR9 | CCAAT/enhancer-binding protein | 26.367297162 | 7.45 |
| A0A7H1KAB2 | Tyrosine-protein kinase | 25.807938169 | 7.11 |
| Q3HP17 | Interleukin enhancer binding factor 2 | 24.95313713 | 5.58 |
| A0A0D3R0W7 | Cytotoxic granule-associated RNA binding protein transcript variant 1 | 24.766330341 | 5.49 |
| A0A0F6MVY1 | NKEF-B | 24.471409784 | 5.37 |
| A0A2H5AC57 | RAB5 | 24.635333748 | 5.30 |
| A0A6G6C405 | cAMP-dependent protein kinase type I-alpha regulatory subunit | 24.212821703 | 5.18 |
| A0A2Z4LXY0 | Runt-related transcription factor | 23.478771594 | 4.95 |
| I4AY76 | Tollip | 23.457011075 | 4.66 |
| F4YAX3 | Heat shock 70 kDa protein | 23.763040502 | 4.58 |
| E7D7N6 | 60 kDa chaperonin | 26.472215846 | 4.51 |
| A0A076PUA2 | Casein kinase II subunit beta (Fragment) | 24.629675896 | 4.45 |
| A0A0K0YD76 | Ras-related C3 botulinum toxin substrate 1 | 24.248438374 | 4.44 |
| C5MR43 | RNA helicase | 24.95282619 | 3.81 |
| A0A1W6AMN5 | NF-kappaB2 | 22.713788397 | 3.67 |
| M4GYA0 | Core binding factor beta | 27.328601386 | 3.35 |
| A0A5B8IM13 | cAMP-dependent protein kinase | 22.396289107 | 3.23 |
| A0A5A4CXJ8 | Ras family member A (Fragment) | 22.62845266 | 3.12 |
| A0A3Q8AG35 | Pyrroline-5-carboxylate reductase | 22.403276295 | 3.05 |
| A0A7T7IN63 | Serine/threonine-protein kinase Nek6 | 22.261352353 | 2.95 |
| Q6E2N6 | Aryl hydrocarbon receptor nuclear translocator 2c | 23.188865887 | 2.89 |
| A0A7G8KPR8 | CCAAT/enhancer-binding protein | 22.26083571 | 2.74 |
| A0A0D5MCQ9 | Histone H2B | 31.22882878 | 2.70 |
| D3JZG0 | SREBP-1 (Fragment) | 21.676246359 | 2.41 |
| C1KH73 | Keratin-like protein (Fragment) | 34.599234546 | 2.16 |
| A0A3G2LZD3 | MKK4 | 21.758411921 | 2.02 |
| R9RIF4 | Voltage-dependent anion-selective channel protein 1 | 28.235942709 | 2.02 |
