# Supplementary material for: A fish-specific E3 ubiquitin ligase NHRE3 suppresses antiviral immunity by degrading ISG15 via ubiquitination
Source: PLoS Pathog. 2026 Sep 29;22(9):e1014544. doi: 10.1371/journal.ppat.1014544 (PMC13623101; doi:10.1371/journal.ppat.1014544)
Supplement: Supplementary file 9 [file ppat.1014544.s008.docx]

**S2 Table. Candidate interacting proteins of NHRE3 in the experimental group**

| **Accession** | **Description** | **log2 intensity** | **log2 FC** |
| --- | --- | --- | --- |
| B3GN92 | Cellular nucleic acid-binding protein | 30.081950538 | 11.26 |
| A0A7G8KPR9 | CCAAT/enhancer-binding protein | 28.286479081 | 9.37 |
| A0A068L824 | Histone H2A (Fragment) | 28.092750851 | 7.98 |
| A0A0D3R0W7 | Cytotoxic granule-associated RNA binding protein transcript variant 1 | 24.949846654 | 5.67 |
| A0A1W6AMN5 | NF-kappaB2 | 24.698958531 | 5.65 |
| A0A7H1KAB2 | Tyrosine-protein kinase | 24.286937286 | 5.59 |
| Q3HP17 | Interleukin enhancer binding factor 2 | 24.888600942 | 5.52 |
| A0A0F6MVY1 | NKEF-B | 24.477907446 | 5.38 |
| A0A2H5AC57 | RAB5 | 24.671524654 | 5.33 |
| F4YAX3 | Heat shock 70 kDa protein | 24.434980914 | 5.25 |
| A0A7U3NG54 | Ubiquitin-like protein 1 | 24.444048316 | 5.11 |
| A0A6G6C405 | cAMP-dependent protein kinase type I-alpha regulatory subunit | 23.840765632 | 4.81 |
| E7D7N6 | 60 kDa chaperonin | 26.094000182 | 4.14 |
| I4AY76 | Tollip | 22.669385103 | 3.88 |
| A0A142G532 | NFkB p65 transcription factor | 23.524802808 | 3.87 |
| M4GYA0 | Core binding factor beta | 27.804889632 | 3.82 |
| A0A076PUA2 | Casein kinase II subunit beta (Fragment) | 23.940109241 | 3.76 |
| A0A3S8Q1S9 | Transcription factor 7 | 22.901317698 | 3.75 |
| A0A7T7IN63 | Serine/threonine-protein kinase Nek6 | 22.828673433 | 3.52 |
| A0A7G8KPR8 | CCAAT/enhancer-binding protein | 22.852966814 | 3.34 |
| Q6E2N6 | Aryl hydrocarbon receptor nuclear translocator 2c | 23.499357366 | 3.20 |
| A0A3Q8AG35 | Pyrroline-5-carboxylate reductase | 22.359201558 | 3.01 |
| A0A5A4CXJ8 | Ras family member A (Fragment) | 22.409375397 | 2.90 |
| Q2PWU2 | Proto-oncogene c-Jun | 22.614905709 | 2.86 |
| A0A0K0YD76 | Ras-related C3 botulinum toxin substrate 1 | 22.522553756 | 2.72 |
| Q2PWU1 | Transcription factor jun-B | 27.81268253 | 2.58 |
| D3JZG0 | SREBP-1 (Fragment) | 21.821119988 | 2.55 |
| A0A3G2LZD3 | MKK4 | 21.962363694 | 2.22 |
| R4L845 | 40S ribosomal protein SA | 26.933258238 | 2.22 |
