# Supplementary material for: A fish-specific E3 ubiquitin ligase NHRE3 suppresses antiviral immunity by degrading ISG15 via ubiquitination
Source: PLoS Pathog. 2026 Sep 29;22(9):e1014544. doi: 10.1371/journal.ppat.1014544 (PMC13623101; doi:10.1371/journal.ppat.1014544)
Supplement: Supplementary file 10 [file ppat.1014544.s009.docx]

**S3 Table. Knockout verification and homologous arm primers**

| **Primers** | **Sequences** |
| --- | --- |
| NHRE3-HA-F | GCGTTTAAACTTAAGCTTATGTACCCATACGATGTTCCAGATTACGCTACTTTCAACGTGGACC |
| NHRE3-HA-R | GCTGGATATCTGCAGAATTCTTATGTTCTCCCAAATTCAG |
| NHRE3_N-HA-F | AGTGTGGTGGAATTCATGTACCCATACGATGTTCCAGATTACGCTACTTTCAACGTGGAC |
| NHRE3_N-HA-R | ACGGGCCCTCTAGACTCGAGTTATGAAGTCAACCGGT |
| NHRE3_C-HA-F | AGTGTGGTGGAATTCATGTACCCATACGATGTTCCAGATTACGCTCTGCAGAGTGCAAAA |
| NHRE3_C-HA-R | ACGGGCCCTCTAGACTCGAGTTATGTTCTCCCAAATTCAG |
| NHRE3-GFP-F | CTAGCGCTACCGGACTCAGATCTCGAGGTATGACTTTCAACGTGGACC |
| NHRE3-GFP-R | GCTCACCATGGTGGCGATGGATCCTGTTCTCCCAAATTCAG |
| ISG15-MYC-F | GGCCCGAATTCGGTCGACCGAGATCTCTGAACTGGCAATAAAACT |
| ISG15-MYC-R | CTTATCATGTCTGGATCCCCGCGGCCGCTTAACCTCCTCGGAGG |
| ISG15-mCherry-F | CGAGCTCAAGCTTCGAATTCATGGAACTGGCAATAAAACT |
| ISG15-mCherry-R | ACCATGGTGGCGATGGATCCACCTCCTCGGAGG |
| IRF3-HA-F | GCGTTTAAACTTAAGCTTATGTACCCATACGATGTTCCAGATTACGCTACCCATCCAAAACCGCT |
| IRF3-HA-R | GCTGGATATCTGCAGAATTCTTACTTGGTGTCACACAACTCC |
| IRF3-GFP-F | CTAGCGCTACCGGACTCAGATCTCGAGGTATGACCCATCCAAAACCGCT |
| IRF3-GFP-R | GCTCACCATGGTGGCGATGGATCCCTTGGTGTCACACAACTCC |
| NS1-F | agtgtggtggaattcatgtacccatacgatgttccagattacgctgcacgccgcattact |
| NS1-R | ACGGGCCCTCTAGACTCGAGTTACAGCAGCAGGGAGG |
| NS3-F | agtgtggtggaattcatgtacccatacgatgttccagattacgctgccaccgcaggaatc |
| NS3-R | ACGGGCCCTCTAGACTCGAGTCATTCAAACACGCACGACA |
| NS5-F | agtgtggtggaattcatgtacccatacgatgttccagattacgctaacgccgaccgca |
| NS5-R | ACGGGCCCTCTAGACTCGAGTTAGCAACCATTGTCCATTAAC |
| *Gr*KO-NHRE3-F | ACCAGAGCAGACACAAGCA |
| *Gr*KO-NHRE3-R | ACCTGAATCAACACCTGCTT |
