# Supplementary material for: A fish-specific E3 ubiquitin ligase NHRE3 suppresses antiviral immunity by degrading ISG15 via ubiquitination
Source: PLoS Pathog. 2026 Sep 29;22(9):e1014544. doi: 10.1371/journal.ppat.1014544 (PMC13623101; doi:10.1371/journal.ppat.1014544)
Supplement: Supplementary file 11 [file ppat.1014544.s010.docx]

**S4 Table. qPCR primers**

| **Primers** | **Sequences** |
| --- | --- |
| Ci-qNHRE3-F | CACCAACATCTGCCCACAAA |
| Ci-qNHRE3-R | TACACCACGCCTACACAAGT |
| Gr-qNHRE3-F | TGGAGTGTCGGACTTGTA |
| Gr-qNHRE3-R | CATCCTCATAACTTGTGGTA |
| Ci-qISG15-F | CGGTTTGCACTCAGGATCAG |
| Ci-qISG15-R | GCCGTTGTAAATCAGTCGCT |
| Ci-qIFN1-F | AAGCAACGAGTCTTTGAGCCT |
| Ci-qIFN1-R | GCGTCCTGGAAATGACACCT |
| Ci-qIFN3-F | TACATTTATAGAGACTGCGGGTGG |
| Ci-qIFN3-R | TGGAGTGTCTGGTAAACAGCCTT |
| Ci-qIRF3-F | TCCAGGCCAAGCATACGAA |
| Ci-qIRF3-R | CCATTTGCAACAGCCATCAT |
| Ci-qIL1β-F | GATTCGAAAGTTCGATTCAATCT |
| Ci-qIL1β-R | TTCAGTGACCTCCTTCAAGAC |
| Ci-qIL6-F | CAGCAGAATGGGGGAGTTATC |
| Ci-qIL6-R | CTCGCAGAGTCTTGACATCCTT |
| Ci-qIL8-R | TCTACCCTCCTAGCCCTCACTG |
| Ci-qIL8-R | TCATGGTGCTTTGTTGGCAAGGA |
| Ci-qMX1-F | CTGGGGAGGAAGTAAAGTGTTCT |
| Ci-qMX1-R | CAGCATGGATTCTGCCTGG |
| Ci-qMX2-F | ACATTGACATCGCCACCACT |
| Ci-qMX2-R | TTCTGACCACCGTCTCCTCC |
| Gr-qβactin-F | GTCCGTGACATCAAAGAG |
| Gr-qβactin-R | ACCGCAAGATTCCATAC |
| Ci-qβactin-F | GACATGGAGAAGATCTGGCA |
| Ci-qβactin-R | CTGTTGGCTTTGGGATTCAG |
| GCRV-qS6-F | CCTGTGCCTCACCTACCCAAA |
| GCRV-qS6-R | GAACCAACGAGACTCCCAACTTC |
| GCRV-qS10-F | CGCACCTGTGACTTGGACGA |
| GCRV-qS10-R | AGCGAGTCAGCACCTTGTCG |
| GCRV-qNS1-F | GTCACTGCCCATTGCTTCGC |
| GCRV-qNS1-R | GCGGCACGGGATCTGTTGTA |
